# Glutamate utilization fuels rapid production of mitochondrial ROS in dendritic cells and drives systemic inflammation during tularemia

**DOI:** 10.1126/sciadv.adu6271

**Published:** 2025-08-29

**Authors:** Ivo Fabrik, Petra Spidlova, Lukas Prchal, Daniela Fabrikova, Ina Viduka, Valentina Marecic, Vlada Filimonenko, Radek Sleha, Marie Vajrychova, Rudolf Kupcik, Ondrej Soukup, Tomas Rousar, Anetta Härtlova, Marina Santic, Jiri Stulik

**Affiliations:** ^1^ Biomedical Research Center, University Hospital Hradec Kralove, 500 05 Hradec Kralove, Czechia.; ^2^ Department of Molecular Pathology and Biology, Military Faculty of Medicine, University of Defence, 500 01 Hradec Kralove, Czechia.; ^3^ Department of Microbiology and Parasitology, Faculty of Medicine, University of Rijeka, 51000 Rijeka, Croatia.; ^4^ Electron Microscopy Core Facility, Institute of Molecular Genetics, Czech Academy of Sciences, 142 20 Prague, Czechia.; ^5^ Laboratory of Biology of the Cell Nucleus, Institute of Molecular Genetics, Czech Academy of Sciences, 142 20 Prague, Czechia.; ^6^ Department of Epidemiology, Military Faculty of Medicine, University of Defence, 500 01 Hradec Kralove, Czechia.; ^7^ Department of Biological and Biochemical Sciences, Faculty of Chemical Technology, University of Pardubice, 532 10 Pardubice, Czechia.; ^8^ Wallenberg Centre for Molecular and Translational Medicine, Department of Microbiology and Immunology at Institute of Biomedicine, University of Gothenburg, 405 30 Gothenburg, Sweden.; ^9^ Institute of Medical Microbiology and Hygiene, Medical Center–University of Freiburg, Faculty of Medicine, 79104 Freiburg, Germany.; ^10^ Department of Environmental Health, Teaching Institute of Public Health of Primorje-Gorski Kotar County, Rijeka, Croatia.

## Abstract

Dendritic cells (DCs) hijacked by intracellular bacteria contribute to pathogen dissemination and immunopathology. How bacteria achieve DC subversion remains largely unknown. Here, we describe the mechanism used by tularemia agent *Francisella tularensis* exploiting host mitochondrial anaplerosis. Shortly after internalization, *Francisella* associates with DC mitochondria, which leads to the rapid repurposing of their oxidative metabolism for production of mitochondrial reactive oxygen species (mtROS). Mitochondrial metabolic rewiring is orchestrated by the intramitochondrial signaling mediated by protein acetylation and involves switching to glutamate as the primary substrate for DC tricarboxylic acid cycle. Rather than killing the bacterium, glutamate-fueled mtROS production activates p38-dependent proinflammatory gene expression. Blocking of glutamate utilization prevents DC activation and bacterial dissemination and alleviates inflammation in vivo. Our findings underscore the importance of metabolic plasticity in antibacterial DC response and open up potential avenues for therapies targeting host metabolism.

## Teaser

Host glutamate feeds activated dendritic cells and fuels bacterial spread and sepsis during tularemia.

## INTRODUCTION

Phagocytes are the first cells that intercept invading pathogens and raise local antimicrobial defense (*1*). Among phagocytes, dendritic cells (DCs) have unique position because their mutual interaction with the microbe initiates the cascade leading to more systemic response (*2*). Upon activation, DCs migrate from the infection site to lymphoid organs where they present antigens to naïve T cells. This induces adaptive immune response that is required for control of infection at the organism level. Inevitably, some pathogens evolved their ways to manipulate DC functions, forcing them into the tolerogenic state (*3*) or causing their aberrant activation that leads to systemic immunopathology (*4*, *5*), as in the case of *Francisella*.

*Francisella tularensis*, the causative agent of tularemia, is a highly infectious Gram-negative intracellular bacterium that invades phagocytes including DCs (*6*). Internalized *Francisella* briefly resides within the maturing phagosome but avoids lysosomal degradation by escaping into the cytosol, where the bacterium proliferates (*7*, *8*). DCs infected by *Francisella* fail to undergo classical maturation (*9*–*13*) but still manage to migrate toward lymph nodes and, therefore, contribute to systemic bacterial dissemination and severe tularemia (*9*, *14*).

The nature of host pathogen interactions responsible for hijacking DC functions by intracellular *Francisella* is unclear. It is generally assumed that phagosomal escape into the cytosol explains the way the bacteria prevent detection by membrane-bound pattern recognition receptors (PRRs) such as Toll-like receptors. Furthermore, *Francisella* lacks many classical PRR ligands (*15*), and virulent strains evade cytosolic inflammasome activation (*11*, *12*) or the autophagic host defense system (*16*, *17*). In contrast to other cytosolic bacteria, *Francisella* does not exploit host actin polymerization (*18*) and secretes only few virulence factors (*19*, *20*), most of which have an unknown function. As such, the bacterium appears unexpectedly inert to the host cell. Recently, *Francisella* was reported to enhance host mitochondrial functions to aid its intracellular replication (*21*, *22*). In addition, two virulence factors secreted by *Francisella* were identified as interaction partners of host mitochondrial proteins (*23*), suggesting that mitochondria might play an essential role in *Francisella* virulence. Manipulation of host mitochondria is a common feature for many intracellular pathogens (*24*–*26*), but the underlying molecular mechanisms are relatively unexplored and even less is known about the impact on DC functions.

Here, we demonstrate that cytosolic *Francisella* in DCs interacts with mitochondria and that this leads to the rapid repurposing of DC mitochondrial oxidative metabolism for production of mitochondrial reactive oxygen species (mtROS). The metabolic reprogramming is coordinated by the intramitochondrial signaling mediated by protein acetylation and involves switching to glutamate as the primary substrate for the tricarboxylic acid cycle (TCA). We show that mtROS production in DCs is proinflammatory and that the restriction of glutamate utilization prevents DC activation and alleviates the rapid progression of severe tularemia.

## RESULTS

### *Francisella* interacts with mitochondria and induces mitochondrial ROS production in DCs

**Fig. 1.**
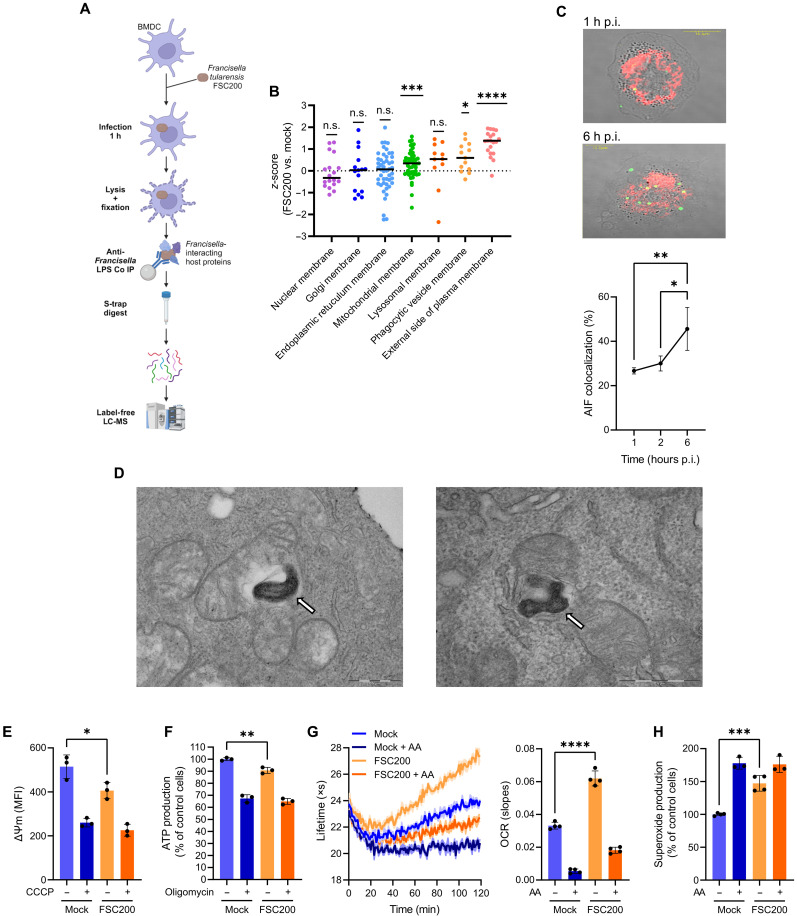
*Francisella* interacts with mitochondria and induces mitochondrial ROS production in DCs. (**A**) Workflow of *Francisella* LPS coimmunoprecipitation (co-IP) experiment. Mock-infected cells were processed identically except for infection. Created in BioRender.com. Gorecki, L. (2025) https://BioRender.com/y05h360. (**B**) BMDC membrane proteins enriched in co-IP samples from *Francisella*-infected cells at 1 hour p.i. based on host subcellular localization according to Gene Ontology (GO). Normalized protein iBAQ ratios were averaged and transformed by *z*-score. n.s., not significant. (**C**) Association of FSC200 (green) with mitochondrial apoptosis-inducing factor (AIF; red) in BMDCs at 1 and 6 hours (h) p.i. analyzed by confocal microscopy (scale bars, 10 μm). Relative quantification of the association is shown in graph below. (**D**) Electron micrographs of cytosolic FSC200 (white arrows) interacting with mitochondria in BMDCs (scale bars, 500 nm). (**E**) Mitochondrial potential in BMDCs infected for 1 hour with FSC200. Pretreatment with 100 μM CCCP for 30 min was used as a control. (**F**) Adenosine 5′-triphosphate (ATP) levels in BMDCs infected for 1 hour with FSC200. Cotreatment with 5 μM oligomycin was used as a control. MFI, median of fluorescent intensity. (**G**) Oxygen consumption rate (OCR) in BMDCs infected for 1 hour with FSC200. Cotreatment with 1 μM antimycin A (AA) was used as a control. (**H**) Superoxide production in BMDCs infected for 1 hour with FSC200. Cotreatment with 1 μM AA was used as a control. Signal response in [(F) and (H)] is normalized to uninfected and untreated cells (=100%). Multiplicity of infection (MOI) was 100. Data in (B) are from one experiment with *n* = 3 biological replicates. Data in [(C) and (F)] are combined from *n* = 3 experiments. Micrographs in [(C) and (D)] and data in [(E) and (G)] are representative from *n* = 3 experiments. Data in (H) are combined from *n* = 4 experiments. Significance was determined by one sample Wilcoxon rank test (B) or one-way analysis of variance (ANOVA) followed by Tukey’s post hoc test. Data are expressed as medians (B) or means ± SD. *P < 0.05; **P < 0.01; ***P < 0.001; ****P < 0.0001.

The intracellular fate of *Francisella* within the host phagosomal/endosomal system shortly after the bacterial internalization [<1 hour postinfection (p.i.)] is relatively well described (*7*). In contrast, little is known about interactions of the bacterium with other host organelles (*27*). To screen for host subcellular components potentially associated with the bacterium, we infected bone marrow–derived DCs (BMDCs) with a fully virulent *F. tularensis* subsp. *holarctica* FSC200 strain and left them for 1 hour when the bacterium escapes from the phagosome (*28*). BMDC lysates were then subjected to anti-*Francisella* lipopolysaccharide (LPS) coimmunoprecipitation (co-IP), and proteins present in co-IP eluates were analyzed by liquid chromatography–mass spectrometry (LC-MS; Fig. 1A). Plasma membrane and phagolysosomal components showed the highest enrichment among host membrane proteins identified in co-IP samples from the infected cells (Fig. 1B and data S1), reflecting a sustained localization of bacteria within the host endosomal system. Mitochondrial membrane proteins were the next most enriched (Fig. 1B), suggesting that mitochondria might also associate with *Francisella*. To confirm the interaction, we analyzed FSC200-infected BMDCs by confocal (Fig. 1C) and electron (Fig. 1D) microscopy starting at 1 hour p.i. More than 25% of internalized bacteria colocalized with host mitochondria at 1 hour p.i. (Fig. 1C). The fraction of mitochondria-associated *Francisella* increased in time (Fig. 1C), suggesting that the interaction occurs also in the cytosol after the phagosomal escape of the bacterium (Fig. 1D). We sought to understand the role of mitochondria in *Francisella* infection, and, hence, our next step was to assess mitochondrial functions in infected DCs. BMDCs were infected with FSC200 for 1 hour and analyzed for changes in mitochondrial potential (Δψ), adenosine 5′-triphosphate (ATP) production, and oxygen consumption. The infection caused a drop in both Δψ and ATP levels (Fig. 1, E and F), indicating the loss of energy-generating functions in mitochondria. However, mitochondrial consumption of oxygen in infected BMDCs increased (Fig. 1G). We speculated that these contradictory findings might be explained by remodeling of the mitochondrial oxidative metabolism toward production of reactive oxygen species (ROS) instead of ATP. To test that, we infected BMDCs with FSC200 for 1 hour and measured their superoxide levels. In line with the previous work (*29*) and our assumption, infected cells exhibited increased superoxide production (Fig. 1H), which was not affected by nicotinamide adenine dinucleotide phosphate oxidase inhibitor apocynin (fig. S1), indicating a mitochondrial origin of the generated ROS (mtROS). Collectively, these data demonstrate that mitochondria in infected DCs interact with intracellular *Francisella* and repurpose their oxidative metabolism for generation of mtROS.

### *Francisella*-induced mtROS activate p38-dependent proinflammatory signaling in DCs

**Fig. 2.**
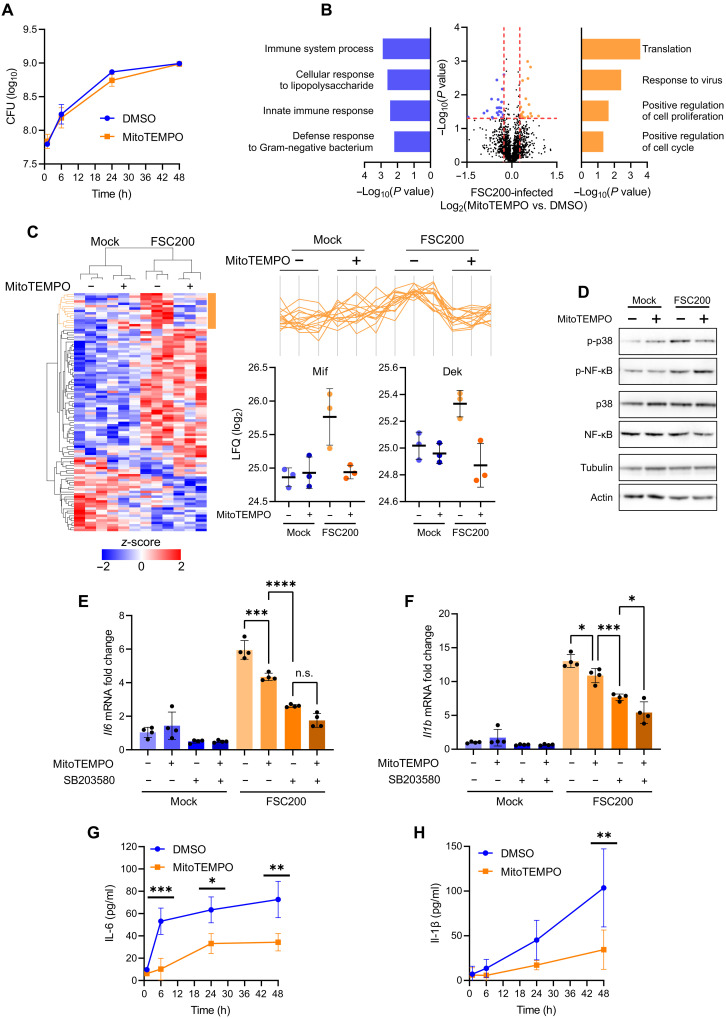
*Francisella*-induced mtROS activate p38-dependent proinflammatory signaling in DCs. (**A**) FSC200 proliferation in BMDC pretreated (or not) with 100 μM MitoTEMPO. h, hours. (**B**) Volcano plot showing proteins differentially expressed in FSC200-infected BMDCs 6 hours p.i. pretreated either with 100 μM MitoTEMPO for 1 hour or left untreated [dimethyl sulfoxide (DMSO)]. GO term enrichment was determined using DAVID (https://davidbioinformatics.nih.gov/). (**C**) Heatmap showing significantly regulated proteins (ANOVA, *P* < 0.05) in BMDCs pretreated (or not) with 100 μM MitoTEMPO and infected (or not) by FSC200 at 6 hours p.i. Orange cluster contains proteins that were up-regulated in infected cells and down-regulated by MitoTEMPO. Log_2_-label-free quantification (LFQ) intensities of selected proteins from orange cluster (Mif and Dek) are shown below. (**D**) p38 and nuclear factor κB (NF-κB) activation in BMDCs pretreated for 1 hour with 100 μM MitoTEMPO and infected with FSC200 (1 hour p.i.). Expression of (**E**) *Il6* and (**F**) *Il1b* in BMDCs pretreated for 1 hour with 100 μM MitoTEMPO or 10 μM SB203580 (p38 inhibitor) and infected with FSC200 (6 hours p.i.). *Actb* was used as a housekeeping gene. n.s., not significant. (**G**) Interleukin-6 (IL-6) and (**H**) IL-1β concentrations in cell culture medium conditioned by BMDCs pretreated (or not) with 100 μM MitoTEMPO for 1 hour and infected with FSC200 for the indicated durations p.i. MOI was 50 [(A) to (C) and (E) to (H)] or 100 (D). Data in [(A) and (D)] are representative from *n* = 3 experiments. Data in [(B) and (C)] are from one experiment with *n* = 3 biological replicates. Data in [(E) and (F)] are combined from *n* = 4 experiments. Data in [(G) and (H)] are combined from *n* = 3 experiments. Significance was determined by *t* test (B) and one-way [(E) and (F)] or two-way[(G) and (H)] ANOVA followed by Tukey’s or Šídák’s post hoc tests, respectively. Data in graphs are expressed as means ± SD. *P < 0.05; **P < 0.01; ***P < 0.001; ****P < 0.0001.

Mitochondrial ROS have been shown to enhance killing of intracellular bacteria in macrophages (*30*). To examine whether *Francisella*-induced mtROS have the same function, BMDCs were first pretreated with mtROS scavenger MitoTEMPO (fig. S2A) and then infected with FSC200 followed by determination of colony-forming units (CFU). Consistent with the previous report (*29*), dampening mtROS levels had no effect on bacterial proliferation (Fig. 2A), suggesting that in our in vitro model mtROS do not contribute to *Francisella* killing. To uncover biologically relevant role of mtROS in *Francisella*-DC interaction, we decided to evaluate the impact of mtROS on host protein expression. The proteome of BMDCs pretreated with MitoTEMPO and infected with FSC200 for 6 hours was compared to the proteome of the same infected cells with unperturbed mtROS levels (Fig. 2B and Data S2). The suppression of mtROS in infected BMDCs caused down-regulation of proteins annotated by Gene Ontology (GO) terms related to innate immune response to bacterium (Fig. 2B and fig. S2B). The down-regulation mediated by MitoTEMPO scavenger was specific to a relatively small fraction of proteins induced by FSC200 infection (Fig. 2C, orange cluster), including secreted factors Mif and Dek (Fig. 2C, graphs), which have been reported to have proinflammatory and chemoattractant functions (*31*, *32*). These data indicate that the major function of mtROS in *Francisella*-infected DCs is to enhance proinflammatory gene expression rather than to kill bacteria. We have previously shown that the early cytokine expression elicited by cytosolic *Francisella* in DCs is dependent on p38 (*28*). Because p38 can be activated by ROS (*33*), we assumed that *Francisella*-induced mtROS are upstream of the p38 signaling cascade. Indeed, quenching of mtROS during the infection suppressed p38 activation (Fig. 2D and fig. S2C), significantly reduced p38-dependent *Il6*, *Il1b*, *Il12a*, and *Il10* expression (Fig. 2, E and F, and fig. S2E) and prevented interleukin-6 (IL-6) and IL-1β secretion (Fig. 2, G and H). The data in (Fig. 2F and fig. S2E) also revealed that blocking of mtROS in p38-inhibited cells had an additional effect on cytokine transcription, indicating the existence of p38-independent mechanism of gene expression regulated by mtROS. One of plausible candidates is the nuclear factor κB (NF-κB) pathway, which is activated in *Francisella*-infected cells (*28*, *34*) and which has been shown to be sensitive to ROS (*35*). Nevertheless, MitoTEMPO treatment had no significant impact on infection-induced p65 phosphorylation (Fig. 2D and fig. S2D), suggesting that NF-κB activation in *Francisella*-infected DCs is regulated independently of mtROS and that other transcription factors, such as activator protein-1 (AP-1) (*28*), may act downstream of p38-independent mtROS signaling. Together, the presented findings demonstrate the key signaling role of mtROS in the proinflammatory activation of *Francisella*-infected DCs and highlight the importance of mitochondrial metabolism in the control of host cytosolic and antibacterial response.

### mtROS production in *Francisella*-infected DCs is Pnpt1-dependent and orchestrated by compartmentalized mitochondrial protein acetylation

Mitochondrial ROS were initially considered as undesired by-products of mitochondrial oxidative metabolism. However, more recent evidence implies that mtROS play an important regulatory role in many aspects of cell biology including cell signaling (*36*). Involvement in such rapid cellular events requires fast adaptation of mitochondrial metabolism to support mtROS production. We observed increased oxygen consumption rate (OCR) and mtROS response in infected DCs within 1 hour p.i. (Fig. 1, G and H), indicating tight and rapid regulation of oxidative metabolism at mitochondrial level.

**Fig. 3.**
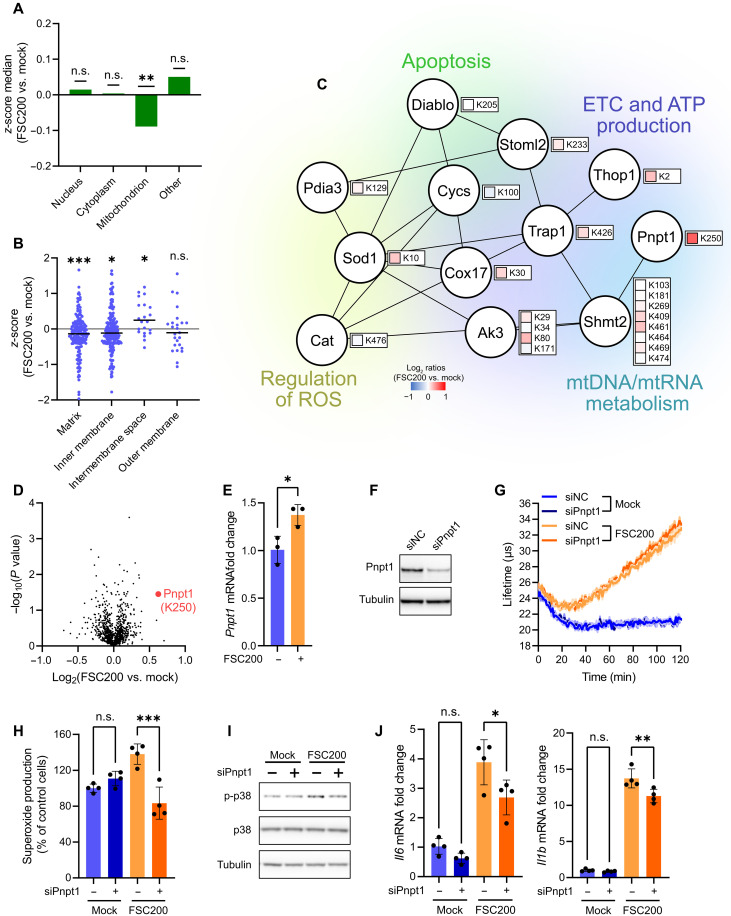
mtROS production in *Francisella*-infected DCs is Pnpt1-dependent and orchestrated by compartmentalized mitochondrial protein acetylation. (**A**) Protein acetylation in BMDCs 1 hour p.i. based on subcellular localization (GO) of modified protein. (**B**) Mitochondrial protein acetylation in BMDCs 1 hour p.i. based on submitochondrial localization (GO) of modified protein. Each dot represents single AcK site. (**C**) STRING protein-protein interaction network of intermembrane space (IMS) proteins containing quantified AcK sites (B). (**D**) Volcano plot showing differentially regulated AcK sites in FSC200-infected BMDCs 1 hour p.i. (**E**) Relative expression of *Pnpt1* in BMDCs infected with FSC200 for 6 hours. *Actb* was used as a housekeeping gene. (**F**) Efficiency of Pnpt1 knockdown (siPnpt1 KD) in BMDCs. Cells transfected by negative control siRNA are used as a control (siNC). (**G**) OCR in siPnpt1 KD BMDCs infected for 1 hour with FSC200. (**H**) Superoxide production in siPnpt1 KD BMDCs infected for 1 hour with FSC200. (**I**) p38 activation in siPnpt1 KD BMDCs infected with FSC200 for 1 hour p.i. (**J**) *Il6* and *Il1b* expression in siPnpt1 KD BMDCs infected with FSC200 for 6 hours p.i. *Actb* was used as a housekeeping gene. Signal response in (H) is normalized to uninfected siNC cells (=100%). MOI was 50 [(A) to (D)] or 100 [(E and (G) to (J)]. Data in [(A) to (D)] are from one experiment and combined from *n* = 2 [(A) to (C)] or *n* = 3 (D) biological replicates. Data in [(F), (G), and (I)] are representative from *n* = 3 experiments. Data in (E) are combined from *n* = 3 experiments. Data in [(H) and (J)] are combined from *n* = 4 experiments. Significance was determined by one sample Wilcoxon rank test [(A) and (B)], *t* test [(D) and (E)], or one-way ANOVA followed by Tukey’s post hoc test [(H) and (J)]. Data are expressed as medians (A) or as means ± SD [(E), (H), and (J)]. *P < 0.05; **P < 0.01; ***P < 0.001. n.s., not significant.

Protein acetylation represents a highly dynamic posttranslational modification (PTM) that controls the activity of metabolic enzymes and complexes in mitochondria (*37*, *38*). For example, it was shown that deacetylation of mitochondrial proteins by SIRT3 deacetylase helped to restore mitochondrial energetic metabolism (*39*). Considering all above, we hypothesized that the rapid (1 hour p.i.) repurposing of mitochondrial metabolism for mtROS production in *Francisella*-infected DCs might be regulated at PTM level by acetylation of key host mitochondrial proteins. To investigate that, we infected stable isotope labeling by amino acids (SILAC)–labeled BMDCs (*40*) with FSC200 for 1 hour and analyzed changes in host protein acetylation by proteomics. Acetylated sites (AcK) from mitochondrial proteins accounted for 34% of all identified AcK sites (fig. S3A and data S3) corroborating the importance of protein acetylation for the regulation of mitochondrial metabolism in DCs. In general, *Francisella* infection caused global deacetylation of proteins in DC mitochondria (Fig. 3A). Specifically, acetylation of proteins located in the mitochondrial matrix and inner membrane (Fig. 3B) and involved in oxidative phosphorylation (OXPHOS; fig. S3B) was down-regulated, suggesting infection-induced activation of SIRT3 deacetylase (fig. S3C) and thus supporting the overall mobilization of mitochondrial oxidative metabolism for mtROS production upon infection (Fig. 1, G and H). We noticed that AcK sites of proteins localized into the mitochondrial intermembrane space (IMS) showed an opposite trend and were up-regulated (Fig. 3B). IMS accumulates protons pumped out from the matrix via components of the electron transport chain (ETC), but it also provides an environment into which a fraction of ETC-generated mtROS is released and dissipated toward the cytosol (*41*). Several IMS proteins with identified AcK sites were involved in control of mtROS homeostasis (Fig. 3C), which prompted us to investigate whether these might regulate mtROS burst upon *Francisella* infection. We turned our attention to Pnpt1, which showed the highest infection-induced change in acetylation (AcK site K250; Fig. 3, C and D) and which was up-regulated at 6 hour p.i. (Fig. 3E). Pnpt1 (or PNPase) is a mitochondrial exoribonuclease localized primarily in IMS and, to a lesser extent, in the matrix (*42*), where it mediates the transport of nucleus-encoded RNA into mitochondria or the degradation of polycistronic dsRNA transcripts, respectively (*43*). Several studies reported that Pnpt1-deficient cells exhibit defects in OXPHOS (*44*, *45*) and that Pnpt1 tunes OXPHOS/glycolysis balance during physiological conditions (*46*), suggesting that Pnpt1 might be involved in the rapid transformation of DC oxidative metabolism, promoting generation of mtROS in response to intracellular *Francisella*. To explore that, we infected BMDCs having Pnpt1 depleted by small interfering RNA (siRNA; Fig. 3F) with FSC200 and analyzed their mitochondrial functions. Silencing of Pnpt1 did not have a significant effect on oxygen consumption in infected cells (Fig. 3G and fig. S3D). However, Pnpt1-deficient DCs generated less mtROS upon infection (Fig. 3H) while keeping their ATP production intact (fig. S3E, compare with Fig. 1F), suggesting that Pnpt1 is required for shifting mitochondrial metabolism from steady-state OXPHOS to mtROS production in infected DCs. Consistently with dampened mtROS response, Pnpt1 deficiency in infected DCs restrained p38 activation (Fig. 3I and fig. S3F) and expression of *Il6*, *Il1b*, and *Il12a* (Fig. 3J and fig. S3G). Together, rapid transformation of mitochondrial metabolism to mtROS production in *Francisella*-infected DCs is tuned by Pnpt1 and orchestrated by sub-mitochondrial signal transduction mediated by protein acetylation.

### *Francisella*-infected DCs fuel their TCA cycle by glutamate catabolism

**Fig. 4.**
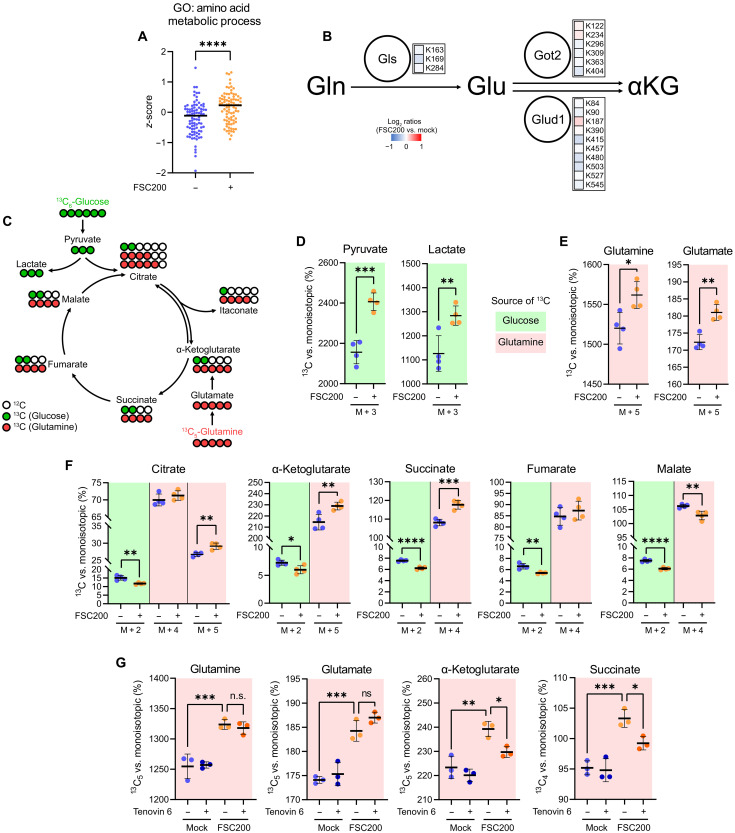
*Francisella*-infected DCs fuel their TCA cycle by glutamate catabolism. (**A**) Expression of proteins annotated by GO term Amino acid metabolic process in BMDCs infected with FSC200 for 6 hours. (**B**) Acetylation of enzymes of glutaminolysis pathway in BMDCs infected with FSC200 for 1 hour p.i. (**C**) Scheme of ^13^C isotope distribution from ^13^C-labeled glucose and glutamine in isotope tracing experiment. (**D**) Increased flux of glucose-originating ^13^C to pyruvate and lactate in FSC200-infected BMDCs at 1 hour p.i. (**E**) Increased uptake of U-^13^C-labeled glutamine and flux of glutamine-originating ^13^C to glutamate in FSC200-infected BMDCs at 1 hour p.i. (**F**) Flux of glucose- and glutamine-originating ^13^C into TCA metabolites in FSC200-infected BMDCs at 1 hour p.i. (**G**) Flux of glutamine-originating ^13^C into glutamate, α-ketoglutarate (αKG), and succinate in FSC200-infected BMDCs cotreated (or not) with 10 μM Tenovin 6 at 1 hour p.i. Background coloring in (D) to (G) refers to the origin of ^13^C (green, glucose; red, glutamine) as indicated in (C). Fluxes in (D) to (F) are expressed as intensity ratios between the indicated isotopic peak (M + X below the *x* axes in graphs) and monoisotopic (unlabeled) peak of the given metabolite. Fluxes in (G) are expressed as intensity ratios between M + 5 for glutamine/glutamate/αKG and M + 4 for succinate and the respective monoisotopic peak. MOI was 50. Data are from one experiment and combined from *n* = 2 [(A) and (B)], *n* = 3 (G), and *n* = 4 [(D) to (F)] biological replicates. Significance was determined by two-sided Mann-Whitney test (A), *t* test [(D) to (F)], or one-way ANOVA followed by Tukey’s post hoc test (G). Data are expressed as medians (A) or as means ± SD [(D) to (G)]. *P < 0.05; **P < 0.01; ***P < 0.001; ****P < 0.0001. n.s., not significant.

Our next aim was to identify metabolic pathways fueling mtROS burst. We noticed that DCs infected by *Francisella* up-regulated proteins related to amino acid metabolism (Fig. 4A). Closer inspection of the mitochondrial acetylome revealed deacetylation of key enzymes of glutamine catabolism toward α-ketoglutarate (αKG; Fig. 4B), suggesting that infected DCs might increase their utilization of glutamine to supply TCA with oxidizable carbon needed for mtROS formation. To examine that, BMDCs incubated with either U-^13^C-labeled glucose or glutamine were infected with FSC200 for 1 hour and then analyzed for ^13^C flux in glycolytic and TCA metabolites (Fig. 4C). *Francisella* infection in DCs stimulated glycolysis as evidenced by the increased fraction of ^13^C-labeled pyruvate (Fig. 4D). However, the increased production of lactate (Fig. 4D) together with the decreased flux of glucose-originating carbon in TCA metabolites (Fig. 4F and fig. S4A) indicates that pyruvate is diverted away from TCA and that glycolysis in infected cells does not drive the formation of mtROS (Fig. 4C). In contrast and in line with our data (Fig. 4, A and B), infected DCs also enhanced their uptake of glutamine and its conversion into glutamate (Fig. 4E), which then entered TCA via αKG (Fig. 4F). Glutamate-fueled TCA appears interrupted and partially reversed, which can be inferred from the decreased ^13^C flux into malate despite succinate accumulation and from the increased carbon flow from αKG to citrate via isocitrate, respectively (Fig. 4F). We then asked how the switch in TCA substrates is regulated. Deacetylation of glutamine-processing enzymes (Fig. 4B) and matrix proteins in general (Fig. 3B) indicated that metabolic enzymes responsible for *Francisella*-induced glutaminolysis are activated by down-regulation of protein acetylation. To examine this, we infected BMDCs labeled by U-^13^C-glutamine with FSC200 and cotreated them with the inhibitor of mitochondrial deacetylation Tenovin 6 (*47*). Deacetylation blockade did not affect glutamine uptake or glutaminase activity, as evidenced by the unperturbed ^13^C flux through glutamine and glutamate in the infected cells (Fig. 4G). However, the incorporation of glutamate-derived carbon into TCA metabolites αKG and succinate was reduced upon Tenovin 6 treatment (Fig. 4G), suggesting that *Francisella*-infected DCs enhance the activity of TCA enzymes by protein deacetylation. These findings were further corroborated by the observation that restricting mitochondrial protein deacetylation in *Francisella*-infected DCs prevented the p38 activation (fig. S4B). Collectively, these results imply that DCs use glutamate catabolism to bolster rapid mtROS production in response to intracellular *Francisella*.

### Got2 drives glutamate-dependent mtROS production and inflammation in *Francisella*-infected DCs

**Fig. 5.**
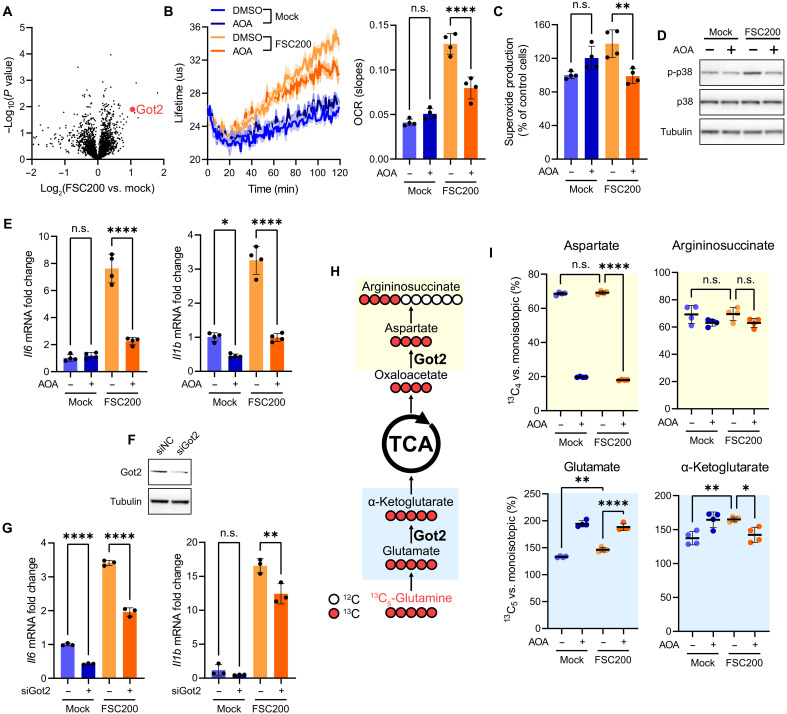
Got2 drives glutamate-dependent mtROS production and inflammation in *Francisella*-infected DCs. (**A**) Volcano plot showing proteins differentially expressed upon FSC200 infection in BMDCs at 6 hours p.i. (**B**) OCR, (**C**) superoxide production, (**D**) p38 activation, and (**E**) *Il6* and *Il1b* expression in BMDCs pretreated (or not) with 10 mM AOA for 1 hour and then infected for 1 hour (B to D) or 6 hours (E) with FSC200. (**F**) Efficiency of Got2 knockdown (siGot2 KD) in BMDCs. (**G**) *Il6* and *Il1b* expression in siGot2 KD BMDCs infected with FSC200 for 6 hours p.i. *Actb* was used as a housekeeping gene (E and G). (**H**) Scheme of ^13^C isotope distribution from ^13^C-labeled glutamine in isotope tracing experiment with AOA. (**I**) Decreased flux of glutamine-originating ^13^C from glutamate to αKG and aspartate upon Got2 inhibition by AOA. U-^13^C-glutamine–labeled BMDCs were pretreated (or not) with 10 mM AOA for 1 hour and then infected for 1 hour with FSC200. Background coloring in (I) refers to the substrate/product pair of Got2-catalyzed reaction as indicated in (H). Fluxes in (I) are expressed as intensity ratios between the respective isotopic peak (M + 5 for glutamate/αKG and M + 4 for aspartate and argininosuccinate) and monoisotopic (unlabeled) peak. Signal response in (C) is normalized to uninfected and untreated cells (=100%). MOI was 50 [(A), (E), (G), and (I)] or 100 [(B) to (D)]. Data in (A) are from one experiment and combined from at least *n* = 2 biological replicates. Data in [(C) and (E)] are combined from *n* = 4 experiments. Data in (G) are combined from *n* = 3 experiments. Data in [(B) and (D)] are representative from *n* = 3 experiments. Data in (I) are from one experiment with *n* = 4 biological replicates. Significance was determined by one-way ANOVA followed by Tukey’s post hoc test. Data in graphs are expressed as means ± SD. *P < 0.05; **P < 0.01; ****P < 0.0001. n.s., not significant.

Metabolism of glutamine and glutamate is controlled by several key enzymes (*48*). One of them, mitochondrial glutamate oxaloacetate transaminase 2 (Got2), was up-regulated in DCs upon *Francisella* infection (Fig. 5A). Got2 mediates entry of glutamate-derived carbon into TCA by catalyzing transamination reaction between glutamate and oxaloacetate to form aspartate and αKG, indicating that glutamate-fueled mtROS production in infected DCs might be critically dependent on Got2 activity. BMDCs pretreated with Got2 inhibitor aminooxyacetic acid (AOA) (*49*) before challenge by FSC200 demonstrated decreased oxygen consumption (Fig. 5B) and superoxide production (Fig. 5C) when compared with untreated cells infected by *Francisella*. Correspondingly, Got2 inhibition restricted p38 activation (Fig. 5D and fig. S5, A and B); suppressed *Il6*, *Il1b*, and *Il12a* expression (Fig. 5E and fig. S5C), IL-6 and IL-1β secretion (fig. S5D), cell surface expression of major histocompatibility complex class II (MHCII), and CD80 (fig. S5E); and improved viability (fig. S5F) in *Francisella*-infected DCs. Furthermore, BMDCs with siRNA-depleted Got2 (Fig. 5F) expressed less *Il6*, *Il1b*, and *Il12a* upon *Francisella* infection (Fig. 5G and fig. S5G), overall confirming the key role of Got2 in regulation of mtROS-mediated inflammatory signaling. We next sought to ascertain which Got2-dependent metabolic pathways contribute to the DC response to *Francisella*. Besides providing TCA with αKG, Got2 also participates in the argininosuccinate shunt by generating aspartate for argininosuccinate synthesis (*49*) and in the malate-aspartate shuttle (*50*). Stable isotope tracing experiment using U-^13^C-glutamine (Fig. 5H) confirmed that AOA treatment in FSC200-infected BMDCs at 1 hour p.i. targets Got2 (*51*, *52*) and leads to the interruption of glutamate-derived carbon flux into TCA (Fig. 5I). This is demonstrated by the decreased formation of αKG and accumulation of glutamate when compared with untreated *Francisella*-infected DCs (Fig. 5I). However, neither infection nor Got2 inhibition significantly affected ^13^C flux into argininosuccinate (Fig. 5I). Similarly, the inhibition of argininosuccinate synthase or 2-oxoglutarate carrier protein did not prevent p38 activation (fig. S5H), together indicating that Got2 drives mtROS production by providing αKG for TCA and not via argininosuccinate shunt or malate-asparate shuttle. Unexpectedly, we also found that pharmacological blockade of glutaminase (fig. S5H) or variation of glutamine concentration in the infection medium (fig. S5I) had only little effect on DC response at early time points p.i., thus strengthening the key role of Got2 and glutamate transamination in DC activation. Last, to address whether *Francisella*-infected DCs also increase their utilization of lipids as an alternative carbon source for mtROS-fueling TCA, we pretreated BMDCs with fatty acid β-oxidation (FAO) inhibitors trimetazidine and etomoxir prior the challenge with FSC200. None of these inhibitors affected mtROS production, p38 activation, or cytokine expression in infected cells (fig. S6, A to C), indicating that, at least in vitro, glutamate serves as the primary substrate fueling proinflammatory TCA cycle. Together, these data underscore the importance of Got2-driven glutamate catabolism in regulating rapid mtROS-mediated inflammatory signaling in DCs exposed to intracellular *Francisella*.

### Inhibition of glutamate transamination in vivo impairs DC trafficking into spleen and attenuates the development of severe tularemia

**Fig. 6.**
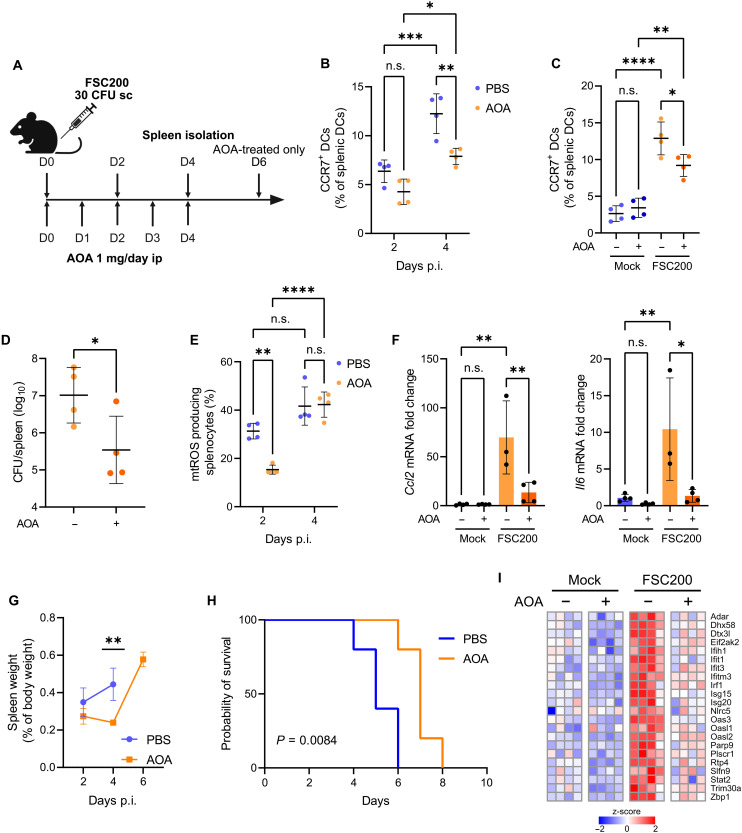
Inhibition of glutamate transamination in vivo impairs DC trafficking into spleen and attenuates the development of severe tularemia. (**A**) Scheme of in vivo experiment. sc, subcutaneous; ip, intraperitoneal; D, day. (**B**) Frequency of migratory DCs (CD11c^+^ MHCII^+^ CCR7^+^) within the population of alive splenic DCs (CD11c^+^ MHCII^+^) in spleens of FSC200-infected mice treated with PBS or AOA on days 2 and 4 p.i. and compared to (**C**) uninfected mice on day 4 p.i. (*n* = 4 mice per group). (**D**) Bacterial loads in spleens of FSC200-infected mice treated with PBS or AOA on day 4 p.i. (*n* = 4 mice per group). (**E**) Splenocyte mtROS production in FSC200-infected mice treated with PBS or AOA on days 2 and 4 p.i. (*n* = 4 mice per group). (**F**) Splenic expression of *Ccl2* and *Il6* in uninfected or FSC200-infected mice treated with PBS or AOA on day 4 p.i. *Tbp* was used as a housekeeping gene (*n* = 3–4 mice per group). (**G**) Spleen weights in FSC200-infected mice treated with PBS or AOA [*n* = 4 mice per group except for day 6 p.i. (AOA), where *n* = 2]. All PBS-treated mice succumbed to infection before day 6 p.i. (**H**) Survival of FSC200-infected mice treated with PBS or AOA (*n* = 5 mice per group). (**I**) Splenic expression of proteins related to host antiviral defense on day 4 p.i. (*n* = 4 mice per group). Data in [(C), (D), (F), and (I)] are from one experiment and data in [(B), (E), and (G)] are from another experiment of similar design (A). Data in (H) are representative from two experiments. Significance was determined by two-sided *t* test (D), one-way ANOVA followed by Tukey’s post hoc test [(C) and (F)], two-way ANOVA followed by Šídák’s post hoc test [(B), (E), and (G)], or log-rank test (H). Data in graphs [(B) to (G)] are expressed as means ± SD. *P < 0.05; **P < 0.01; ***P < 0.001; ****P < 0.0001. n.s., not significant.

We next investigated whether the inhibition of glutamate transamination in the host could protect from severe infection in vivo. Lethal tularemia is characterized by hypercytokinemia and tissue damage, which results from uncontrolled bacterial growth and dissemination during the initial phase of the infection (*53*). DCs are among the first cells to be infected by *Francisella* in vivo (*9*), mediating its spread by migration through lymphatics (*14*). We reasoned that, if glutamate-fueled mtROS production in DCs is unable to limit bacterial intracellular proliferation (Fig. 2A), then the resulting DC activation (Fig. 5, E and G; and fig. S5, C to G) would rather contribute to excessive inflammation and bacterial dissemination. Supporting this hypothesis, our in vitro experiments with a well-established model of migratory DC (*54*) Flt3L-derived BMDCs (fig. S7A) indicated that *Francisella*-infected cells use glutamate not only to drive mtROS burst and cytokine expression (fig. S7, B and C) but also to up-regulate the lymph node-homing receptor CCR7 (fig. S7D). Restricting glutamate metabolism may, therefore, limit inflammation and bacterial spread and could be beneficial for the host. To address this, we subcutaneously inoculated C57BL/6 mice with FSC200 and administered them phosphate-buffered saline (PBS; mock) or AOA daily for five days, starting on the day of infection. Mice were euthanized on indicated days and their spleens collected and processed (Fig. 6A). Although all infected mice exhibited the increased splenic infiltration of migratory CCR7^+^ DCs (fig. S7E) (*55*) from day 2 to day 4 p.i. (Fig. 6B), AOA treatment significantly attenuated this progression by day 4 (Fig. 6, B and C, and fig. S7F) without affecting lymphocyte populations (fig. S7G). Correspondingly, bacterial loads were lower in spleens of AOA-treated mice (Fig. 6D), indicating that the inhibition of glutamate transamination in the host suppresses DC trafficking and bacterial dissemination. In line with in vitro data (Fig. 5C and figs. S5F and S7B), AOA improved splenocyte viability (fig. S7H) and suppressed splenic mtROS production (Fig. 6E and fig. S7I) in infected mice, although both effects were observable on day 2 p.i. and thus probably unrelated to infiltrating DCs (Fig. 6B). AOA administration also alleviated splenic expression of *Ccl2*, *Il6*, or *Il10* (Fig. 6F and fig. S7J), which was induced during the infection. Notably, higher levels of CCL2 and IL-6 in spleen are markers of lethal tularemia (*56*), suggesting that the utilization of glutamate might contribute to host sepsis and death. Indeed, AOA treatment delayed the onset and reduced the development of infection-induced splenomegaly (Fig. 6G) and improved the survival of infected mice (Fig. 6H). Notably, AOA delayed mortality also in BALB/c mice (fig. S7K), which are more resistant to *Francisella* and exhibit prolonged survival compared to C57BL/6 mice (*57*). To further investigate the impact of AOA administration on cellular level, we analyzed protein expression in splenocytes isolated from *Francisella*-infected AOA-treated C57BL/6 mice on day 4 p.i. and compared them to the control groups (fig. S8A and data S4). AOA treatment in general up-regulated proteins related to carbohydrate metabolism and glycolysis (fig. S8A, cluster C1, and fig. S8B) while reduced levels of TCA components (fig. S8A, cluster C2, and fig. S8C), indicating that AOA diminishes metabolic flux through mitochondria in splenic cells. In infected mice, AOA specifically suppressed the expression of proteins involved in the antiviral response (Fig. 6I and fig. S8A, cluster C3), which are implicated in translational shutdown and organ injury during the late phase of bacterial sepsis (*58*). Accordingly, symptoms of severe tularemia on day 4 p.i. were less pronounced in AOA-treated mice (Fig. 6G), indicating that the host antiviral response contributes to severe tularemia and that the inhibition of glutamate transamination alleviates the rapid progression of systemic disease.

## DISCUSSION

Various bacterial or viral pathogens induce mtROS production as a part of the innate immune host defense (*59*). The toxic action of mtROS helps to limit pathogen growth and the resulting oxidative stress can mediate expression of proinflammatory genes (*30*). However, uncontrolled mtROS production may lead to sepsis and tissue damage detrimental to the host (*60*). Consequently, much effort was put into the understanding of mechanisms governing formation of mtROS to prevent related harmful effects. On the basis of results from the *Francisella* infection model, we show here that mtROS production in infected cells is critically dependent on the utilization of glutamate.

The presented data support the model in which mitochondria-generated ROS promote DC activation in response to intracellular pathogen. We demonstrate that DC mitochondria associate with intracellular bacteria and rapidly, within 1 hour p.i., reprogram their oxidative metabolism toward mtROS production, which, in turn, activates p38 and early proinflammatory gene expression. Furthermore, we show that this is accompanied by switching to glutamate as the primary TCA substrate driving mtROS and that the inhibition of the key glutamate-transaminating enzyme Got2 in infected DCs prevents mtROS formation and proinflammatory cytokine expression. Activated DCs mediate the development of antibacterial adaptive response (*61*), but they also contribute to bacteria-induced sepsis (*62*). Consequently, inflammatory DCs, which are unable to control bacterial proliferation, serve rather as Trojan horses disseminating pathogen (*63*) with the potential to induce excessive inflammation. Our data show that administration of a small molecule inhibitor blocking glutamate utilization in vivo prevents DC trafficking and bacterial dissemination, attenuates the onset of severe infection, and improves survival.

Previous work of Crane and colleagues has demonstrated that mtROS are required for AIM2 inflammasome-mediated IL-1β secretion in *Francisella*-infected macrophages (*29*). Our data are in agreement with these findings as dampening mtROS levels restricted IL-1β release. Moreover, we show that mtROS promote p38-dependent *Il1b* transcription, suggesting that mitochondrial metabolism in *Francisella*-infected cells regulates both the expression of the IL-1β precursor and its cleavage into the mature form. It has been proposed that mtROS do not activate inflammasome directly but instead promote bacterial damage that leads to the leakage of bacterial DNA into the cytosol, which is, in turn, sensed by AIM2 (*8*, *29*). The magnitude of such response would, therefore, depend on the sensitivity of bacteria to ROS. Virulent *Francisella* strains known to be resistant to oxidative stress evade the activation of inflammasome, as demonstrated for highly virulent *F. tularensis* subsp. *tularensis* Schu S4 (*29*). Although *F. tularensis* subsp. *holarctica* FSC200 used throughout this study is a virulent strain derived from clinical isolate (*64*), it is more sensitive to ROS compared to Schu S4 (*65*, *66*), which likely explains the observed IL-1β secretion in FSC200-infected DCs.

It is well accepted that mtROS are formed at sites associated with ETC or substrate catabolism (*67*), although less is known about the mechanisms regulating mtROS production itself. Our investigation suggests that the rapid generation of mtROS in infected DCs is controlled by the mitochondrion-intrinsic signal propagation via protein acetylation. Global deacetylation of matrix proteins observed upon infection enhanced glutamate flux via TCA and correlated with the early activation of SIRT3 deacetylase, which is known to mobilize mitochondrial enzymes involved in oxidative metabolism including TCA and FAO (*39*). The detected shift in deacetylation of glutamine and glutamate utilizing enzymes suggests that SIRT3 helps to coordinate the TCA substrate switch required for mtROS production. Somewhat contradictorily, SIRT3 activity has been also reported to protect from mtROS by deacetylating superoxide dismutase 2 (*68*). However, such SIRT3-mediated protection might be limited to homeostatic conditions, where mtROS are generated by basal electron leakage during OXPHOS, or to pathological states associated with hypoxia (*69*). In contrast, *Francisella*-induced increase in mitochondrial oxygen consumption and glutamate processing via TCA indicates that the rapid mtROS production in infected DCs is a controlled process benefiting from SIRT3 activity. Transformation of TCA hallmarked by the buildup of glutamate-derived succinate in infected DCs resembles metabolic rewiring in macrophages activated by LPS (*70*, *71*) where the enhanced glutaminolysis supports succinate accumulation. Unlike LPS-stimulated macrophages, however, infected DCs did not augment the formation of argininosuccinate (*49*) or antibacterial itaconate (*72*), indicating that the main function of rewired TCA remains the production of electron donors for mtROS.

The formation of mtROS in infected DCs likely occurs at ETC via reversed electron transport sustained by oxidation of accumulated succinate (*71*). In addition, we have identified IMS exonuclease Pnpt1 as an important factor required for mtROS boost. In infected DCs, Pnpt1 deficiency hindered mtROS production, yet it did not affect oxygen consumption, suggesting that Pnpt1 bolsters mtROS-generating functions of ETC downstream of the glutamate metabolic switch. ETC complexes are known to rapidly modify their architecture upon sensing of bacteria to adjust immune response (*73*). Pnpt1 localized in IMS has been reported to support ETC functions (*44*), and Pnpt1 acetylation observed in our data indicates immediate regulation of such activity. Nevertheless, the role of protein acetylation in IMS of infected DCs remains obscure, as well as whether it is enzymatic or not. Because acetyl-CoA cannot cross the mitochondrial inner membrane directly, the most plausible source of acetyl group for protein modification would be cytosolic citrate (*74*), indicating that processes controlling mitochondrial protein acetylation in the matrix and in the IMS are different and potentially unrelated. Correspondingly, molecular mechanisms behind Pnpt1-dependent metabolic remodeling are not fully understood and remain a subject of research (*45*).

The crucial step in herein-described glutamate-based anaplerosis of TCA repurposed for mtROS production is glutamate transamination catalyzed by Got2. Pharmacological blockade of this reaction by AOA inhibitor not only suppressed mtROS production and activation in infected DCs in vitro but also was associated with a delayed onset of splenic mtROS and a slower accumulation of migratory DCs in the spleen during bacteria-induced sepsis in vivo. Our data do not allow us to definitively conclude whether the reduced DC trafficking reflects impaired DC migration or diminished recruitment. Although AOA treatment down-regulated the expression of CCR7 in infected DCs in vitro, AOA administration in vivo may suppress the production of CCR7 ligands, CCL19 and CCL21, by splenic stromal cells (*75*), potentially contributing to the observed phenotype. Indeed, AOA administration likely affects other immune (*52*) or nonimmune cells (*76*). Nevertheless, the increased glutamate utilization seems to be a common hallmark of infected phagocytes (*77*, *78*), indicating that these innate immune effectors are particularly sensitive to such metabolic intervention. The presented finding that blocking of host glutamate-dependent mitochondrial metabolism can ameliorate the onset of severe bacterial infection, therefore, provides potential perspectives for therapies targeting the early stages of immune response.

## MATERIALS AND METHODS

### Mice

C57BL/6NCrl and BALB/c mice (Velaz, Czechia) were housed in specific pathogen–free conditions with access to food and water ad libitum. All experiments using mice were performed in accordance with guidelines of the Animal Care and Use Ethical Committee of the Military Faculty of Medicine, University of Defence, Czech Republic (project no. 121890/2021-1457). Except for the survival analysis shown in fig. S7K, all in vivo and in vitro experiments were done using C57BL/6NCrl mice or their bone marrow progenitors, respectively.

### Inhibitors and reagents

The following chemicals were used for the treatment throughout the study: carbonyl cyanide 3-chlorophenylhydrazone (CCCP; catalog no. C2759, Merck), oligomycin (catalog no. O4876, Merck), antimycin A (AA; catalog no. A8674, Merck), MitoTEMPO (catalog no. SML0737, Merck), SB203580 (catalog no. S8307, Merck), AOA (catalog no. HY-107994, MedChemExpress), apocynin (catalog no. 178385, Merck), bis-2-(5-phenylacetamido-1,3,4-thiadiazol-2-yl)ethyl sulfide (BPTES, catalog no. HY-12683, MedChemExpress), R162 (catalog no. HY-103096, MedChemExpress), α-methyl-dl-aspartic acid (catalog no. HY-W142119, MedChemExpress), phenylsuccinic acid (catalog no. P35200, Merck), Tenovin 6 (catalog no. 13086, Cayman chemicals), Trimetazidine (catalog no. HY-B0968A, MedChemExpress), and Etomoxir (catalog no. HY-50202, MedChemExpress).

### Primary BMDCs

BMDCs were generated from bone marrow progenitors isolated from femurs and tibias of 6- to 8-week-old female C57BL/6NCrl mice. Unless otherwise noted, bone marrow cells were seeded into a bacterial plastic petri dish (day 0) with 10 ml of RPMI 1640 medium containing 10% (v/v) heat-inactivated fetal bovine serum (FBS; Gibco) and 5% (v/v) supernatant from Ag8653 cells transfected by cDNA of murine granulocyte-macrophage colony-stimulating factor and cultivated at 37°C in a humidified atmosphere of 5% CO_2_. Additional 5 and 2.5 ml of fresh medium were added on days 3 and 6, respectively. Suspension cells were collected on day 8. Flt3L-derived BMDCs were generated by cultivating bone marrow progenitors with murine recombinant Flt3L (100 ng/ml; 427-FL-025, R&D Systems) for 8 days with replacing half of medium with fresh on days 3 and 6.

### SILAC of BMDCs

Metabolic labeling of BMDCs by SILAC was done according to previously published protocol (*40*) with slight modifications. Briefly, BMDCs were generated using SILAC RPMI 1640 (Thermo Fisher Scientific) supplemented with light proline and either light or isotopically labeled arginine (^13^C_6_) and lysine (^13^C_6_, ^15^N_2_). Concentrations of dialyzed FBS (Merck) and Ag8653 supernatant were 10% and 5% (v/v), respectively.

### siRNA knockdown in BMDCs

BMDCs were transfected by Pnpt1 FlexiTube siRNA (Mm_Pnpt1_4, GeneGlobe ID: SI01383032, QIAGEN), by Got2 FlexiTube siRNA (Mm_Got2_2, GeneGlobe ID: SI01054970), or by Negative Control siRNA (QIAGEN). First, 20 μl of the respective 20 μM stock siRNA or 80 μl of HiPerFect transfection reagent (QIAGEN) was added into 500 μl of OptiMEM (Gibco). These two mixtures were combined (1 ml), left for 15 min, and added dropwise to 1.8 × 10^7^ BMDCs resuspended in 2 ml of 50% BMDC-conditioned medium on a 10-cm petri dish. Additional 6 ml of 50% BMDC-conditioned medium were added after 6 hours, and BMDCs were left for another 18 hours at 37°C/5% CO_2_. BMDCs were then seeded into fresh 50% BMDC-conditioned medium and left for another 24 hours before performing experiments.

### Cultivation of bacteria and infections

*F. tularensis* subsp. *holarctica* strain FSC200 was cultured on McLeod agar enriched for bovine hemoglobin and Iso-Vitalex (both Becton Dickinson) at 37°C. Unless otherwise noted, all infection experiments were done as described (*28*): Harvested BMDCs were seeded into fresh RPMI 1640 medium containing 10% FBS at 1 × 10^7^ cells/ml. Infection was initiated by the addition of bacteria suspended in the medium of the same composition followed by thorough mixing. Multiplicity of infection (MOI) was 50 or 100.

### Co-IP of *Francisella*-interacting host proteins

BMDCs (5 × 10^7^ per treatment group) were either infected at MOI of 100 or left untreated (mock) for 1 hour at 37°C/5% CO_2_. After the infection, cell suspensions were diluted by the addition of excess of ice-cold PBS and centrifuged (400*g*/5 min/4°C). BMDC pellets were then washed twice with ice-cold PBS and once with ice-cold 10 mM Hepes. Cells were then resuspended in 0.7 ml of ice-cold 10 mM Hepes and lysed by Dounce homogenizer. Lysates were mixed with 0.7 ml of 4% paraformaldehyde (PFA) and left for 1 hour at room temperature (RT), followed by quenching of PFA by the addition of 70 μl of 20× Tris-buffered saline (TBS). Lysates were then centrifuged (50*g*, 2 min), and the supernatants were transferred into new tubes and centrifuged (4000*g*, 5 min). Pellets were washed twice with TBS and resuspended in TBS containing inhibitors of proteases (Roche). Protein concentration was determined by bicinchoninic acid (BCA) assay (Merck), and equal amounts of protein material for each sample were transferred into new tubes and precleared by Control Agarose beads (Pierce Classic IP Kit, Thermo Fisher Scientific) for 1 hour at 4°C on upside-down rotator. Samples from both mock- and FSC200-infected cells were then let stand for 5 min on ice, and supernatants were transferred into new tubes, mixed with 5 μl of anti-*Francisella* LPS monoclonal antibody (catalog no. ab2033, Abcam), and incubated on upside-down rotator at 4°C overnight. Immunoprecipitates were then transferred into tubes with Protein A/G beads (Pierce Classic IP Kit, Thermo Fisher Scientific) and left for 1 hour on upside-down rotator at RT. Beads were briefly centrifuged (1000*g*, 10 s) and washed four times with TBS and once with Conditioning Buffer (Pierce Classic IP Kit, Thermo Fisher Scientific). Proteins were eluted with 50 μl of 5% SDS/50 mM triethylammonium bicarbonate (TEAB) and digested by S-trap (Protifi). Briefly, samples were reduced with 10 mM tris(2-carboxyethyl)phosphine (TCEP) for 30 min at RT, alkylated by 10 mM IAA for 30 min in the dark, acidified with 27.5% phosphoric acid, and mixed with 90% methanol (MeOH)/100 mM TEAB. Samples were then loaded into S-trap columns and digested with 1 μg of trypsin/Lys-C mixture (Promega) at 37°C overnight. Peptides were eluted by consecutive washes of 50 mM TEAB, 0.2% formic acid (FA), and 50% acetonitrile (ACN) and dried in SpeedVac.

### Measurement of mitochondrial potential

BMDCs were infected with FSC200 (MOI of 100) and treated with dimethyl sulfoxide (DMSO) or 100 μM CCCP for 30 min. Cells were then stained by 500 nM MitoTracker Orange CM-H2TMRos (catalog no. M7511, Thermo Fisher Scientific) and left for another 30 min at 37°C/5% CO_2_. At 1 hour p.i., cell suspensions were diluted by the addition of excess of ice-cold PBS, centrifuged (400*g*/5 min/4°C), and pellets were washed once more with ice-cold PBS. Cells were fixed with 2% PFA for 30 min on ice in the dark followed by washing with PBS, and the medians of fluorescent intensity were analyzed on Guava easyCyte (Merck).

### Measurement of ATP

ATP levels were determined by CellTiter-Glo Luminescent Cell Viability Assay (catalog no. G7571, Promega). BMDCs were infected by FSC200 and treated with DMSO or 5 μM oligomycin for 1 hour. BMDC suspensions corresponding to 1.5 × 10^4^ cells were transferred to a 96-well plate and lysed by CellTiter-Glo Reagent for 10 min. Luminescence within range of 445 to 665 nm was measured on Spark plate reader (Tecan) and normalized to uninfected cells.

### Measurement of OCR

OCR was analyzed by Extracellular Oxygen Consumption Assay (catalog no. ab197243, Abcam). BMDCs infected by FSC200 for 1 hour were seeded into black 96-well plate at a density 2 × 10^5^ per well in 150 μl of fresh medium (with DMSO or 1 μM AA when indicated). Next, 10 μl of OCR reagent was added, and wells were overlaid by two drops of mineral oil. Fluorescence kinetics were measured on Spark plate reader (Tecan) using excitation/emission (Ex/Em) of 380 nm/650 nm (width 20 nm) by dual read time-resolved fluorescence with delay time of 30 and 70 μs for the first (*F1*) and second (*F2*) read, respectively. Integration time was 30 μs, and fluorescence was measured continuously in 1-min steps. Lifetime was calculated as (70 − 30)/ln(*F2*/*F1*). OCR rates were determined as slopes of the signal during the linear phase characterized by the change of lifetime versus time (minutes).

### Measurement of superoxide production

BMDCs were resuspended in Dulbecco’s modified Eagle’s medium/F-12 medium without phenol red (Gibco) and infected by FSC200 (MOI of 100) for 1 hour. Cells were then stained by 5 μM MitoSOX Red (catalog no. M36007, Thermo Fisher Scientific) for 10 min at 37°C in the dark. After staining, cell suspensions were diluted by the addition of excess of Hanks’ balanced salt solution (HBSS) and centrifuged (400*g*/5 min/4°C), and pellets were washed once more by ice-cold HBSS. Stained BMDCs were resuspended in HBSS and transferred into a black 96-well plate at a density of 5 × 10^5^ per well, and fluorescence was measured at Ex/Em of 396 nm/610 nm. Fluorescence signals were corrected by subtraction of blanks (wells without cells) and normalized to protein content in cell lysates from each sample determined by BCA assay (Merck). For measurement of mtROS production in splenocytes, isolated cells were stained as described above and analyzed on Guava easyCyte (Merck) using blue laser and yellow channel (Ex and Em of 488 nm and 583/26 nm, respectively). Unstained cells were used as a negative control for setting the gate.

### Bacterial proliferation (CFU)

BMDCs were pretreated (or not) with 100 μM MitoTEMPO for 1 hour and infected with FSC200 (MOI of 50). After 30 min of incubation at 37°C/5% CO_2_, extracellular bacteria were killed using gentamicin (5 μg/ml). At specific time points (1, 6, 24, and 48 hours) p.i., cells were lysed with 0.1% sodium deoxycholate (SDC), and the lysates were plated on McLeod agar plates at appropriate dilutions. The plates were incubated at 37°C for 5 to 6 days. The number of viable intracellular bacteria was determined by counting the CFU. To analyze the number of FSC200 bacteria in spleen tissues of FSC200-infected mice treated (or not) with AOA, splenocytes resuspended in PBS were plated on McLeod agar plates supplemented with penicillin G (100 U/ml) at appropriate dilutions. The number of viable intracellular bacteria was determined by CFU counts after 6 days of incubation at 37°C.

### Analysis of BMDC proteome

BMDCs were pretreated (or not) with 100 μM MitoTEMPO for 1 hour, infected (or not) by FSC200 (MOI of 50) and kept for 6 hours at 37°C/5% CO_2_. After the infection, cell suspensions were diluted by the addition of excess of ice-cold PBS and centrifuged (400*g*/5 min/4°C). Pellets containing 3 × 10^6^ cells were washed once more by ice-cold PBS and lysed in 2.5% SDC (w/v)/100 mM TEAB with Benzonase (Merck). Protein concentrations in lysates were determined by BCA assay (Merck), and 50 μg from each sample was reduced, alkylated, and digested as described for acetylome analysis. Digests were desalted using Peptide Desalting Spin Columns (Thermo Fisher Scientific) and dried in SpeedVac.

### Proteomic analysis of splenocytes

Splenocytes isolated on day 4 p.i. were lysed with lysis buffer (8 M urea/1% SDC/100 mM TEAB) and centrifuged. Supernatants were transferred into new tubes, and protein content was determined by BCA assay (Merck). Protein amounts corresponding to 100 μg were reduced by 5 mM TCEP at 37°C for 60 min, alkylated by 20 mM CAA at RT for 30 min in the dark, and precipitated by addition of 6 volumes of prechilled acetone at −20°C overnight. Precipitates were centrifuged, and pellets were dissolved in 100 mM TEAB. Proteins were digested by Lys-C/trypsin mixture (Promega) at protein-to-enzymes ratio 25:1 (w/w) at 37°C overnight. Peptides were quantified by Fluorescent Peptide Assay (catalog no. 23290, Thermo Fisher Scientific), and equal amounts from each sample were labeled by TMTpro 16plex (catalog no. A44522, lot: ZJ391848, Thermo Fisher Scientific). Labeled samples were combined into the multiplex and fractionated into eight fractions using the High-pH Reversed-Phase Peptide Fractionation Kit (catalog no. 84868, Thermo Fisher Scientific) and dried in SpeedVac.

### qRT-PCR analysis

Isolation of RNA from BMDCs and splenocytes was done using the RNeasy Mini Kit (QIAGEN) according to the manufacturer’s instructions. RNA was reverse transcribed by the High-Capacity cDNA Reverse Transcription Kit (Thermo Fisher Scientific), and reverse transcription–quantitative polymerase chain reaction (qRT-PCR) analysis was done on LightCycler 96 (Roche) using TaqMan Universal Master Mix II (Thermo Fisher Scientific). The following murine TaqMan probes (Thermo Fisher Scientific) were used: *Il1b* (Mm00434228_m1), *Il6* (Mm00446190_m1), *Il10* (Mm01288386_m1), *Il12a* (Mm00434169_m1), *Il12b* (Mm01288989_m1), *Ccl2* (Mm00441242_m1), *Pnpt1* (Mm00466286_m1), and *Tnf* (Mm00443258_m1). The expression levels were normalized to *Actb* (Mm02619580_g1) or *Tbp* (Mm01277042_m1) using delta-delta cycle threshold (ΔΔCt) method.

### Enzyme-linked immunosorbent assay (ELISA)

Levels of IL-6 and IL-1β in culture supernatants were measured by DuoSet ELISA kits (DY406-05 and DY401-05, R&D Systems) according to the manufacturer’s instructions.

### Flow cytometry

BMDCs or isolated splenocytes at a density of 1 × 10^6^ per mouse were stained either with Fixable Viability Stain 575 V or 780 (catalog no. 565694 or 565388, BD Biosciences) for 15 min on ice in the dark according to the manufacturer’s instructions and washed by fluorescence-activated cell sorting (FACS) buffer (2% FBS in PBS). Fc receptors were blocked by Fc Receptor Binding Inhibitor Polyclonal Antibody (catalog no. 14-9161-73, Thermo Fisher Scientific) used at 100 μg/ml for 20 min on ice in dark. Cells were stained with anti-mouse CD11c-APC (catalog no. 550261, BD Biosciences), MHCII I-A[b]-FITC (catalog no. 553551, BD Biosciences), CCR7-BV421 (catalog no. 562675, BD Biosciences), CD86-PE (catalog no.553692, BD Biosciences), CD3-FITC (catalog no. 555274, BD Biosciences), and CD19-BV421 (catalog no. 562701, BD Biosciences) using dilution of 1:200 for 30 min on ice in dark. Cells were then washed with FACS buffer, fixed with 4% PFA for 20 min on ice in dark, resuspended in FACS buffer, and analyzed on Guava easyCyte (Merck).

### In vivo experiments

C57BL/6NCrl or BALB/c mice (four to five mice per group, 6 to 10 weeks old) were subcutaneously infected with 200 μl of FSC200 suspension (≈30 CFU per mouse). A control group of mice was inoculated with physiological saline solution. Mice were then intraperitoneally injected with PBS or AOA (1 mg per mouse, pH 6 to 7, five doses) every day starting on the day of infection (day 0). For analysis of spleens, mice were euthanized either on day 2, day 4, or day 6 as indicated in the respective experiments. Isolated spleens were homogenized in 70-μm Nylon cell strainers (Corning), and erythrocytes were removed by lysis in red blood cell lysis buffer (Gibco). Splenocytes were resuspended in PBS and used as described in the respective assay section.

### Analysis of acetylome

#### Infection, lysis, and protein digestion

In SILAC experiments, mock-treated BMDCs labeled by one SILAC channel served as a control. Harvested SILAC-labeled BMDCs were seeded into the respective SILAC medium with 10% dialyzed FBS, and one group of SILAC cells was infected (MOI of 50) as described above. Cells were kept at 37°C/5% CO_2_ for 1 hour. After the infection, cell suspensions were diluted by the addition of excess of ice-cold PBS and centrifuged (400*g*/5 min/4°C). Pellets containing 10 × 10^7^ to 12 × 10^7^ SILAC-labeled BMDCs were washed once more by ice-cold PBS and lysed in 2.5% SDC (w/v)/50 mM NH_4_HCO_3_ and heated to 95°C for 5 min in water bath. Lysates were cooled down on ice, treated with Benzonase (Sigma-Aldrich) for 1 hour, and diluted by 50 mM NH_4_HCO_3_ to achieve 1% concentration of SDC. Protein concentrations were measured by BCA kit (Sigma-Aldrich), and corresponding light and heavy SILAC-labeled lysates were mixed in a 1:1 ratio based on protein content (typical total amount of protein after mixing for one biological replicate was 20 to 25 mg). Proteins were reduced by the addition of dithiothreitol (DTT; final concentration, 10 mM) for 1 hour at 37°C, followed by the alkylation with iodoacetamide (IAA; final concentration, 20 mM) for 30 min at RT in the dark. Excess of IAA was quenched by the addition of DTT to a final concentration of 20 mM, and the reaction was left to proceed for 15 min at RT. Proteins were digested by trypsin (Promega) at a ratio 50:1 (w/w) at 37°C overnight. Digestion was stopped by the addition of trifluoroacetic acid (TFA) to a final concentration of 1% (v/v) to precipitate SDC. The suspension was then mixed with an equal volume of ethyl acetate, vortexed, and centrifuged. The upper organic phase was removed, and the extraction process was repeated twice to completely extract SDC. The water phase containing peptides was desalted on Discovery DSC-18 SPE cartridges (500 mg/3 ml; Sigma-Aldrich). The eluate in 60% ACN/0.1% TFA was frozen at −80°C overnight and lyophilized for at least two days to remove TFA.

#### High-pH peptide fractionation

Lyophilized peptides were dissolved in 20 mM ammonium formate (NH_4_FA) containing 2% ACN, pH 10 (mobile phase A). Peptide material corresponding to 5 to 8 mg (one-fourth of one biological replicate) was injected onto a ZORBAX 300 Extend-C18 column (4.6 mm by 250 mm, 5 μm) with guard column (4.6 mm by 12.5 mm, 5 μm; both Agilent) under conditions of 100% of mobile phase A at a flow rate of 0.5 ml/min using Ultimate 3000 binary RSLC system (Thermo Fisher Scientific). Peptide separation was performed by a linear gradient formed by mobile phase A and 80% ACN, 20 mM NH_4_FA, and pH 10 (mobile phase B) from 0 to 50% of mobile phase B in 40 min. Through the gradient elution window, 40 fractions (1 min each) were manually collected into 10 falcon tubes by pooling fractions in 10 min intervals (e.g., fractions 1, 11, 21, and 31). The fractionation was repeated four times for each lyophilized sample to process all peptide material (see above), and the respective fractions were collected and pooled into the same falcon tubes (resulting in 10 fractions each containing ~2 to 2.5 mg of peptides). A small aliquot from each pooled fraction was kept for proteome analysis; the rest was frozen at −80°C overnight and lyophilized for 3 days.

#### Immunoaffinity enrichment of acetylated peptides

Enrichment of lysine-acetylated (AcK) peptides from lyophilized high-pH (HpH) fractions was done using the PTMScan Acetyl-Lysine Motif [Ac-K] Kit (Cell Signaling Technology) by dividing immunoaffinity beads from one vial into 10 immunoaffinity purification (IAP) buffer-dissolved fractions of the respective biological replicate. Subsequent steps were done as described in the protocol supplied by Cell Signaling Technology. AcK peptides from the fractions were desalted using 3 M Empore C18 disks (Sigma-Aldrich) packed into pipette tips, eluted by 60% ACN/0.1% TFA, and dried up in SpeedVac.

### LC-MS analysis of peptides

Ultimate 3000 RSLCnano system connected through Nanospray Flex ion source either with Q Exactive Plus or Orbitrap Exploris 480 equipped with FAIMS Pro Duo interface (Thermo Fisher Scientific) was used for proteomic analyses. Peptides were first introduced onto the trap column (PepMap100 C18, 3 μm, 0.075 mm by 20 mm) and then separated by gradient of 2% ACN/0.1% FA (mobile phase A) and 80% ACN/0.1% FA (mobile phase B) on the analytical column. AcK peptides from HpH fractions were separated by running a gradient from 2 to 34.5% B in 45 min and from 34.5 to 45% B in 5 min at a flow rate of 250 nl/min on PepMap C18 column (2 μm, 0.075 mm by 250 mm). Peptides from BMDC proteome and co-IP samples were separated using the same conditions as for AcK-enriched HpH fractions except for a longer gradient running from 2 to 9% B in 57 min, from 9 to 34.5% B in 160 min, and from 34.5 to 45% B in 23 min. TMTpro-labeled fractions of splenocyte proteome were separated by running gradient from 2 to 34.5% B in 70 min, from 34.5 to 45% B in 10 min, and from 45 to 90% B in 1 min. Mass spectra from AcK-enriched HpH fractions were acquired using by Q Exactive Plus using full mass spectrometry (MS)/Top10 setup. The positive ion MS spectra from 300 to 1700 mass/charge ratio (*m*/*z*) range were obtained at a resolution of 70,000. Multiply charged precursor ions with a minimal threshold intensity of 2.5 × 10^4^ counts and not fragmented during the previous 20 s were admitted for higher-energy collisional dissociation (HCD). Tandem mass spectra were acquired with the following settings: resolution at 35,000, automatic gain control (AGC) target value at 1 × 10^6^, maximum ion injection time at 120 ms, and normalized collision energy (NCE) set to 28. Fixed first mass was set to 100 *m*/*z*. BMDC proteome and co-IP samples were acquired by Q Exactive Plus using MS/Top12 setup. The positive ion MS spectra from 350 to 1600 *m*/*z* range were obtained at a resolution of 70,000. Multiply charged precursors ions with a minimal threshold intensity of 1 × 10^5^ counts and not fragmented during the previous 20 s were admitted for HCD. MS/MS spectra were acquired with the following settings; resolution at 17,500, AGC target value at 1 × 10^6^, maximum ion injection time at 120 ms, NCE set to 28, and without fixed first mass. TMTpro-labeled fractions of splenocyte proteome were acquired by Orbitrap Exploris 480 using MS/Top10 setup. The positive ion MS spectra from 350 to 1400 *m*/*z* range were obtained at a resolution of 60,000. MS precursors above 5000 threshold were isolated using 1.3 *m*/*z* isolation window and fragmented by HCD at NCE of 35. Dynamic exclusion was set to 17 s. MS/MS spectra were acquired at a resolution of 15,000 using AGC target 200% and maximum injection time of 34 ms with fixed first mass of 110 *m*/*z*. TurboTMT feature was enabled. FAIMS Pro Duo was operated under a gas flow of 4.6 liters/min, with compensation voltages set at −45 and −60 V.

### Proteomic data search and quantification

Proteomic datasets, except for TMTpro-labeled fractions of splenocyte proteome (see below), were processed by MaxQuant (versions 1.6.5.0 and 1.6.14.0) coupled to Andromeda search engine (*79*). Data were searched against a reference proteome for *Mus musculus* (UP000000589) downloaded from UniProt site. MaxQuant-implemented database was used for the identification of contaminants. False discovery rate (FDR) estimation of peptide identification was based on target-decoy approach using reverted search database as a decoy. MaxQuant parameters for processing of data from AcK-enriched samples were as follows: mass tolerance for the first search, 20 parts per million (ppm); for the main search from recalibrated spectra, 4.5 ppm (with individual mass error filtering enabled); maximum of three missed cleavages; maximal charge per peptide, *z* = 7; minimal length of peptide, seven amino acids; maximal mass of peptide, 4600 Da; and carbamidomethylation (C) as fixed and oxidation (M) and acetylation (K and protein N-term) as variable modifications with the maximum number of variable modifications per peptide set to 5. Trypsin with no cleavage restriction was set as a protease. Mass tolerance for fragments in MS/MS was 20 ppm, taking the 12 most intensive peaks per 100 Da for search (with enabled possibility of cofragmented peptide identification). Minimal Andromeda score for modified peptides was 40, and minimal delta score for modified peptides was 6. FDR filtering on peptide spectrum match was 0.01 with separate FDR filtering for each modification set to 0.01. For peptide quantitation in SILAC-labeled samples, Arg + 6 [^13^C_6_] and Lys + 8 [^13^C_6_, ^15^N_2_] were set as labels in heavy channel with requantify function enabled. AcK site ratios in a given replicate were calculated as ratios between AcK site signals from infected cells versus mock-treated cells normalized by subtraction of the log ratios’ median and corrected for changes in expression of the respective protein. MaxQuant parameters for processing of proteome and co-IP data were kept as above except for a maximum number of two missed cleavages allowed and only oxidation (M) and acetylation (protein N-term) set as variable modifications. All hits identified in searches as contaminants were filtered out. For protein quantitation in SILAC-labeled samples (acetylome), only protein groups with at least one unique or razor peptide having SILAC ratio and only those protein groups passing protein FDR filtering set to 0.01 were considered. Proteome samples (6 hours p.i.) were quantified using MaxLFQ algorithm requiring at least one ratio of razor or unique peptide for pairwise comparisons of protein abundance between samples. Only protein groups with at least one unique peptide and only those protein groups passing FDR filtering set to 0.01 were considered. Match between runs was enabled with a match time window of 0.7 min and alignment time window of 20 min. Quantification in co-IP samples was done using Intensity-Based Absolute Quantification (iBAQ) values. Only protein groups with at least one unique peptide and only those protein groups passing FDR filtering set to 0.01 were considered. Match between runs was enabled with a match time window of 0.7 min and alignment time window of 20 min. For each replicate, hits identified as immunoglobins were removed, and iBAQ intensities of remaining proteins were normalized so that medians of log_2_ ratios between FSC200- and mock-infected samples are 0. Normalized ratios were then used for further data interpretation. TMTpro-labeled splenocyte proteome data were interpreted using MSFragger (version 4.1) (*80*) integrated into FragPipe (version 22.0). The setting was based on “TMT16” workflow with slight modifications. Precursor mass tolerance was 20 ppm, and fragment mass tolerance was 20 ppm with mass recalibration and parameter optimization enabled. Trypsin was set as a protease with two allowed missed cleavages, and minimal peptide length was seven amino acids. Methionine oxidation (+15.9949), protein N terminus acetylation (+42.0106), TMTpro modification of peptide N terminus (+304.20715), and clipping of N-terminal methionine were set as variable modifications. Carbamidomethylation of cysteine (+57.02146) and TMTpro modification of lysine (+304.20715) were set as fixed modifications. MS/MS spectra were searched against reference proteome of *M. musculus* downloaded from UniProt (UP000000589; 19 April 2025) combined with FragPipe-supplied contaminants and decoy sequences. Peptide-spectrum matches (PSMs) were validated by Percolator (version 3.06.5), and ProteinProphet and Philosopher (version 5.1.1) were used for protein inference and filtering of proteins at 1% FDR. Quantitation was carried out by TMT-Integrator (version 5.0.9) taking razor and unique peptides with minimal PSM probability of 0.9 and data were normalized by median centering.

### Western blot

Denatured and reduced proteins were separated on NuPAGE 4 to 12% Bis-Tris Gels (Thermo Fisher Scientific) and transferred to 0.45-μm polyvinylidene difluoride membranes. Blots were blocked by milk and incubated with primary antibody overnight followed by secondary antibody conjugated with horseradish peroxidase (Thermo Fisher Scientific). Bands were visualized by enhanced chemiluminescence (ECL, Amersham) and captured by Azure c280 (Azure Biosystems). The following primary antibodies were used: anti-tubulin (catalog no. ab6046, Abcam), anti–p-p38 (catalog no. 4511, Cell Signaling Technology), anti-p38 (catalog no. 8690, Cell Signaling Technology), anti-Pnpt1 (catalog no. ab157109, Abcam), anti–p-NF-κB p65 (catalog no. 3033, Cell Signaling Technology), anti–NF-κB p65 (catalog no. 8242, Cell Signaling Technology), anti-Got2 (catalog no. 39627, Cell Signaling Technology), and anti–β-actin (catalog no. ab8226, Abcam). Densitometry was done by using ImageJ (version 1.53k), and band densities were normalized to maximal summed intensity, loading control, and the total level of the respective protein.

### Confocal laser scanning microscopy

BMDCs were infected at MOI of 100. At 1 and 6 hours p.i., the cells were washed with PBS, fixed using 4% PFA (Sigma-Aldrich) for 30 min at 4°C, and permeabilized with 0.5% Triton X-100 (Sigma-Aldrich). The coverslips were incubated with 3% bovine serum albumin (Sigma-Aldrich) for 45 min. Mouse monoclonal anti-*Francisella* LPS antibodies (catalog no. ab2033, Abcam, UK) and rabbit antibody against mitochondrial apoptosis-inducing factor (Cell Signaling Technology) were added for 1 hour at RT. Samples were washed with PBS and incubated with anti-mouse Alexa Fluor 488 (Invitrogen) and anti-rabbit Alexa Fluor 555 (Invitrogen) secondary antibodies for 1 hour at RT. All samples were mounted in Mowiol 4-88 (Sigma-Aldrich), and analyses were performed on FV 1000 Olympus confocal microscope. The results for each experiment were obtained by counting three independent slides each for at least 100 cells.

### Transmission electron microscopy

Infected BMDCs were quickly washed with Sörensen buffer (SB; 0.1 M sodium/potassium phosphate buffer, pH 7.3) at 37°C, fixed with 2.5% glutaraldehyde in SB for 2 hours, washed with SB, embedded in blocks of 1% low-melting point agarose (type VII, Sigma-Aldrich), and postfixed with 1% OsO_4_ solution in SB for 2 hours. Cells were dehydrated in series of ethanol washes with increasing concentration of solvent and subsequently in propyleneoxide and embedded in Epon-Durcupan resin. Polymerized blocks were cut into 80-nm ultrathin sections, collected on 200 mesh size copper grids, and stained with saturated aqueous solution of uranyl acetate for 4 min. The sections were examined in FEI Morgagni 268 transmission electron microscope operated at 80 kV. The images were captured using Mega View III CCD camera (Olympus Soft Imaging Solutions).

### Measurement of SIRT3 activity

SIRT3 activity was measured by the SIRT3 Activity Assay Kit (catalog no. ab156067, Abcam) according to the manufacturer’s instructions. Briefly, all assay reagents were added into PureGrade microtiter plate (Brand) and mixed well. The reactions were initiated by adding 5 μl of BMDC lysates or radioimmunoprecipitation assay lysis buffer and thorough mixing. The fluorescence intensities were read for 60 min at 1-min intervals using FLUOstar OPTIMA (BMG Labtech) microtiter plate fluorometer with excitation at 355 nm and emission at 460 nm.

### Isotope tracing experiments and LC-MS analysis of metabolites

BMDCs were transferred into RPMI medium without glucose or without glutamine (both Gibco) containing 10% dialyzed FBS and ^13^C_6_ glucose (2 g/liter) or 200 mM ^13^C_5_ glutamine (Cambridge Isotope Laboratories), respectively, and incubated for 2 hours at 37°C/5%. Cells were then infected with FSC200 (MOI of 50) for 1 hour. After the infection, cell suspension were diluted by the excess of ice-cold PBS and centrifuged (400*g*/5 min/4°C). Pellets corresponding to 5 × 10^6^ cells were washed once more with ice-cold PBS and lysed in 500 μl of 80% MeOH (−20°C). Lysates were centrifuged (14,000*g*, 10 min, 4°C), and supernatants were directly injected into LC-MS. Mass spectra were obtained using Dionex Ultimate 3000 UHPLC coupled to Q Exactive Plus mass spectrometer (Thermo Fisher Scientific, Waltham, MA, USA) in hydrophilic interaction liquid chromatography mode. Samples were separated using an amide column (Acquity BEH Amide, 2.1 mm by 150 mm, 1.7 μm, Waters) with VanGuard (BEH Amide 1.7 μm 2.1 mm by 5 mm, Waters, Milford, MA, USA) guard column with 10 mM ammonium acetate buffer (pH 10) as mobile phase A and ACN as mobile phase B. The flow was constant at 0.35 ml/min, and the column was kept at 35°C. The method started with 0.5 min of isocratic flow of 5% A, and, then, the gradient of A rose to 60% B in 7.5 min and remained constant at 60% A for 2 min. The composition then reverted to 5% A and equilibrated for 5 min. Total run time of the method was 15 min. Sample injection was 5 μl. Detection was performed by mass spectrometry in negative mode. Settings of the heated electrospray source were as follows: spray voltage, 2.5 kV, capillary temperature, 262°C; sheath gas, 50 arbitrary units; auxiliary gas, 12.5 arbitrary units; spare gas, 2.5 arbitrary units; probe heater temperature, 425°C; max spray current, 100 μA; and S-lens radio frequency (RF) level, 50. MS spectra were collected as Total ion current in the range of 70 to 750 *m*/*z* with resolution 140,000. Acquired spectra were processed by Xcalibur Qual Browser and Xcalibur Quan Browser (Thermo Fisher Scientific, Waltham, MA, USA) using the standards of selected metabolites (Merck, Darmstadt, Germany). ^13^C isotope masses were calculated using ChemDraw software (PerkinElmer, Shelton, CT, USA). For a given analyte, the peak area under the curve of the respective isotopic peak was extracted and averaged from two injections (technical replicates), and metabolic flux was expressed in % as the intensity ratio between the respective isotopic variant and the monoisotopic form.

### Statistical analysis

Statistical analyses were done using GraphPad Prism version 9.3.0, Perseus version 1.6.2.1, or R. Comparison between groups was done by two-sided Student’s *t* test, Mann-Whitney test, and one-way or two-way analysis of variance (ANOVA) followed by Tukey’s or Šídák’s post hoc tests, respectively. Comparison against hypothetical value was done by one sample *t* test or Wilcoxon signed-rank test. Survival curves were compared by log-rank (Mantel-Cox) test.
