# Supplementary material for: Glutamate utilization fuels rapid production of mitochondrial ROS in dendritic cells and drives systemic inflammation during tularemia
Source: Sci Adv. 2025 Aug 29;11(35):eadu6271. doi: 10.1126/sciadv.adu6271 (PMC12396335; doi:10.1126/sciadv.adu6271)
Supplement: Supplementary file 1 — Figs. S1 to S8 Legends for data S1 to S5 [file sciadv.adu6271_sm.pdf]

Science Advances
AAAS

Supplementary Materials for

Glutamate utilization fuels rapid production of mitochondrial ROS in dendritic cells and drives systemic inflammation during tularemia

Ivo Fabrik *et al.*

Corresponding author: Ivo Fabrik, ivo.fabrik@fnhk.cz; Jiri Stulik, jiri.stulik@unob.cz

Sci. Adv. **11**, eadu6271 (2025)
DOI: 10.1126/sciadv.adu6271

The PDF file includes:

Figs. S1 to S8
Legends for data S1 to S5

Other Supplementary Material for this manuscript includes the following:

Data S1 to S5

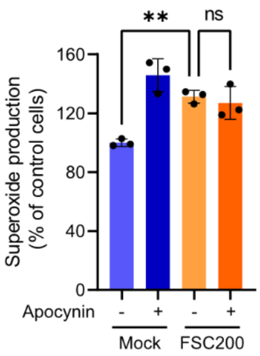

Fig. S1. NADPH oxidase does not contribute to superoxide production in *Francisella*-infected DCs. Superoxide production in BMDCs pretreated (or not) with 100 μM apocynin for 1h and then infected for 1h with FSC200. Signal response is normalized to uninfected and untreated cells (=100%). Multiplicity of infection was 100. Data are representative from n=2 experiments. Significance was determined by one-way ANOVA followed by Tukey's post hoc test. Data in graph are expressed as means ± SD. **P< 0.01.

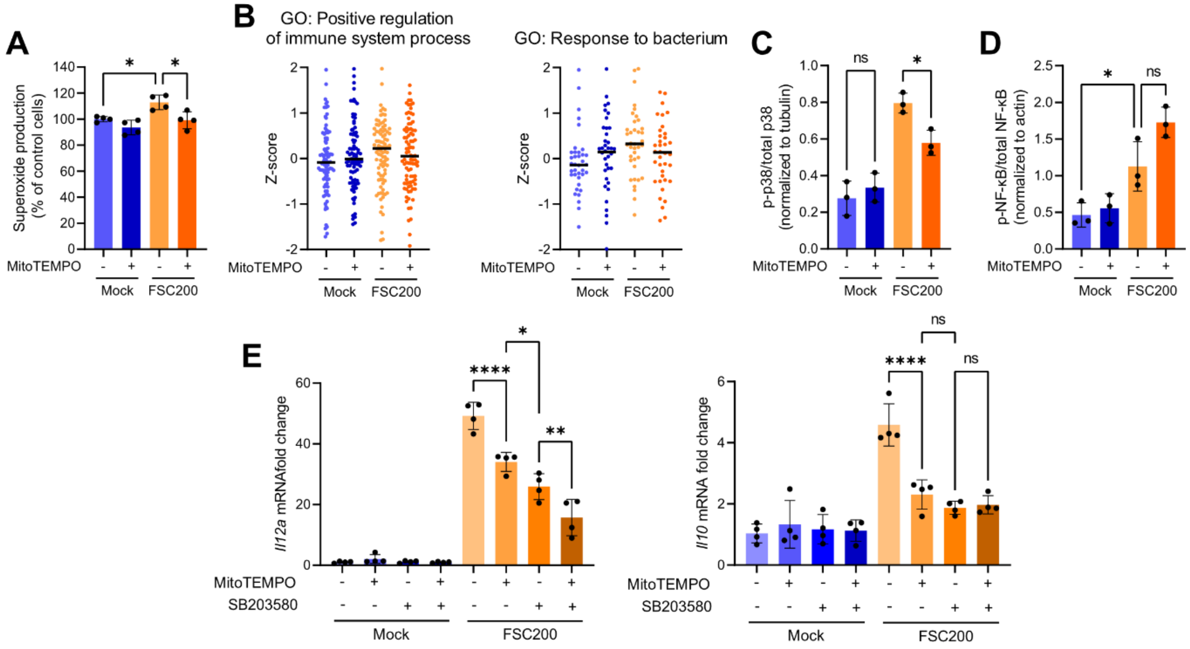

Fig. S2. *Francisella*-induced mtROS control the expression of proinflammatory genes in DCs. (**A**) Superoxide production in BMDCs pretreated (or not) with 100 μM MitoTEMPO for 1h and then infected for 1h with FSC200. (**B**) Expression of proteins annotated by GO terms Positive regulation of immune system process and Response to bacterium in BMDCs infected with FSC200 for 6h. LFQ values of proteins quantified in all 3 replicates were averaged and transformed by Z-score. Each dot represents one protein. Black horizontal lines correspond to medians. Upregulation or downregulation of protein expression is indicated by Z>0 or Z<0, respectively. Densitometric analysis of (**C**) p38 and (**D**) NF-κB activation in **Fig. 2D**. Blot signals were normalized to maximal summed intensity, tubulin (**C**) or actin (**D**) loading control, and total levels of p38 and NF-κB, respectively. (**E**) Expression of *Il12a* and *Il10* in BMDCs pretreated for 1h with 100 μM MitoTEMPO or 10 μM SB203580 (p38 inhibitor) and infected with FSC200 (6h p.i.). *Actb* was used as a housekeeping gene. Signal response in (**A**) is normalized to uninfected and untreated cells (=100%). Multiplicity of infection was 50 (**B**, **E**) or 100 (**A**, **C**, **D**). Data in (**A**) are representative from n=2 experiments. Data in (**B**) are from one experiment from at least n=2 biological replicates. Data in (**C**, **D**) are combined from n=3 experiments. Data in (**E**) are combined from n=4 experiments. Significance was determined by one-way ANOVA followed by Tukey's post hoc test. Data are expressed as medians (**B**) or as means ± SD. *P< 0.05, **P< 0.01, ***P< 0.001, ****P<0.0001.

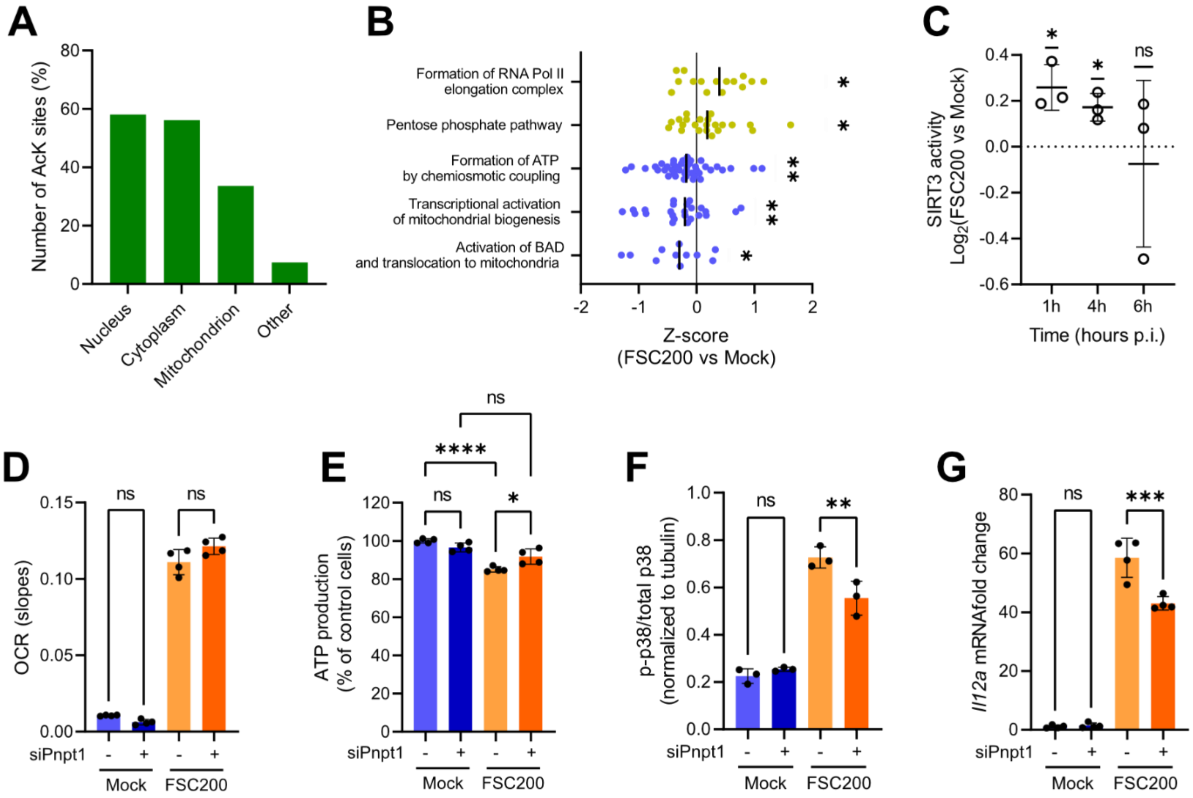

Fig. S3. ***Francisella*** **induces deacetylation of mitochondrial proteins and Pnpt1-dependent drop of ATP in infected DCs.** (**A**) Percentage of all identified AcK sites annotated by the respective GO term. Note that some sites were annotated by several terms simultaneously. (**B**) Protein acetylation in BMDCs 1h p.i. based on Reactome annotation of modified protein. SILAC ratios (FSC200-infected versus Mock-infected cells) of AcK sites quantified in at least 2 replicates were averaged and transformed by Z-score. Each dot represents single AcK site. Black horizontal lines correspond to medians. Upregulation or downregulation of protein acetylation is indicated by Z>0 or Z<0, respectively. Only significant Reactome terms are shown. (**C**) SIRT3 activity in FSC200-infected BMDCs during 1-6h p.i. (**D**) OCR slopes from siPnpt1 KD BMDCs infected for 1h. Related to **Fig. 3G**. (**E**) ATP levels in siPnpt1 KD BMDCs infected for 1h with FSC200. (**F**) Densitometric analysis of p38 activation in **Fig. 3I**. Blot signals were normalized to maximal summed intensity, tubulin loading control, and total levels of p38. (**G**) *Il12a* expression in siPnpt1 KD BMDCs infected with FSC200 for 6h p.i. *Actb* was used as a housekeeping gene. Signal response in (**E**) is normalized to uninfected and untreated cells (=100%). Multiplicity of infection was 50 (**A**, **B**, **C**, **G**) or 100 (**D**, **E**, **F**). Data in (**A**, **B**) are from one experiment with at least n=2 biological replicates. Data in (**C**, **F**) are combined from n=3 experiments. Data in (**D**) are representative from n=3 experiments. Data in (**E**, **G**) are combined from n=4 experiments. Significance was determined by one sample Wilcoxon rank test (**B**), one sample t-test (**C**), or one-way ANOVA followed by Tukey's post hoc test (**D**, **E**, **F**, **G**). Data are expressed as medians (**B**) or means ± SD (**C**, **D**, **E**, **F**, **G**). *P< 0.05, **P< 0.01, ***P< 0.001, ****P<0.0001.

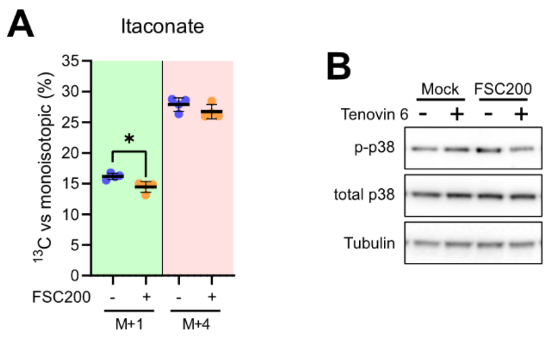

Fig. S4. *Francisella*-infected DCs rewire their glutamate metabolism. (**A**) Flux of glucose- and glutamine-originating ^{13}C into itaconate in FSC200-infected BMDCs at 1h p.i. Background coloring refers to the origin of ^{13}C (green = glucose, red = glutamine) as indicated in **Fig. 4C**. (**B**) p38 activation in FSC200-infected BMDCs co-treated (or not) with 10 µM Tenovin 6 at 1h p.i. Fluxes in (**A**) are expressed as intensity ratios between the indicated isotopic peak (M+X below *x* axis in graph) and monoisotopic (unlabeled) peak of the metabolite. Multiplicity of infection was 50 (**A**) or 100 (**B**). Data in (**A**) are from one experiment with n=4 biological replicates. Data in (**B**) are representative from n=2 experiments. Significance was determined by two-sided t-test. Data are expressed as means ± SD. *P< 0.05.

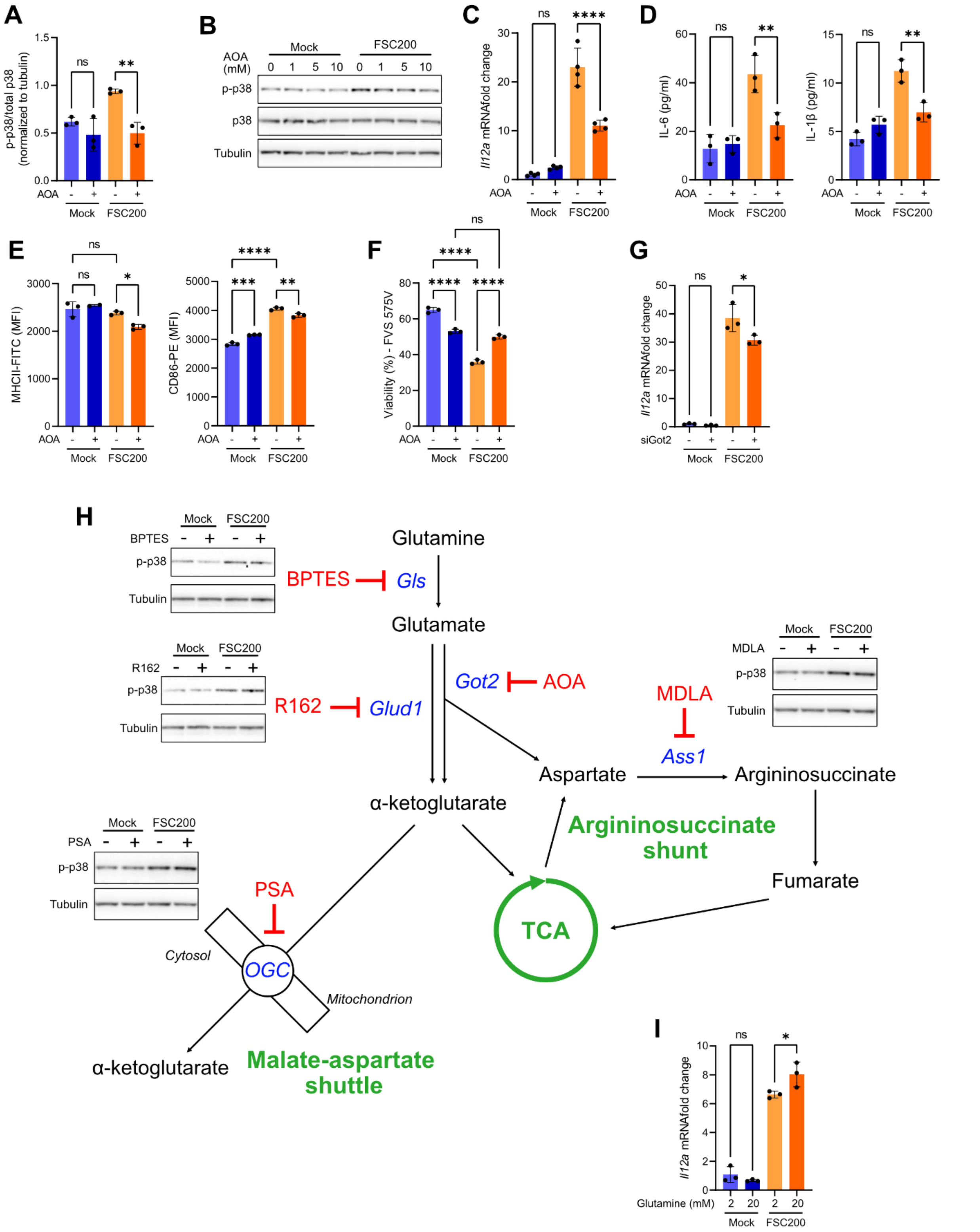

Fig. S5. Got2 activity is required for glutamate-driven activation in *Francisella*-infected DCs. (**A**) Densitometric analysis of p38 activation in **Fig. 5D**. Blot signals were normalized to maximal

summed intensity, tubulin loading control, and total levels of p38. (**B**) p38 activation in BMDCs pretreated (or not) with 1 mM, 5 mM, or 10 mM AOA for 1h and then infected for 1h with FSC200. (**C**) *Il12a* expression in BMDCs pretreated (or not) with 10 mM AOA for 1h and then infected for 6h with FSC200. *Actb* was used as a housekeeping gene. (**D**) Concentration of IL-6 and IL-1β in cell culture medium conditioned by BMDCs which were pretreated (or not) with 10 mM AOA for 1h and then infected with FSC200. IL-6 and IL-1β levels were measured 6h and 24h p.i., respectively (see also **Fig. 2G** and **2H**). (**E**) Cell surface expression of MHCII and CD86 and (**F**) viability of BMDCs pretreated (or not) with 10 mM AOA for 1h and then infected for 24h with FSC200. (**G**) *Il12a* expression in siGot2 KD BMDCs infected with FSC200 for 6h p.i. *Actb* was used as a housekeeping gene. (**H**) Scheme of metabolic pathways related to glutamate/α-ketoglutarate metabolism. Selected inhibitors (red) were used to probe the importance of the respective enzymatic reactions (enzymes/transporters in blue) for activation of p38 in BMDCs infected with *Francisella*. BMDCs pretreated (or not) with 10 μM BPTES (glutaminase inhibitor), 25 μM R162 (glutamate dehydrogenase 1 inhibitor), 10 mM PSA (2-oxoglutarate carrier protein inhibitor), or 5 mM MDLA (argininosuccinate synthase inhibitor) and then infected for 1h with FSC200. Inhibition of p38 activation by AOA is presented in **Fig. 5D** and in (**B**). (**I**) *Il12a* expression in BMDCs preincubated in medium with varying concentration of glutamine (2 and 20 mM, without GM-CSF) for 24h and then infected for 6h with FSC200. *Actb* was used as housekeeping gene. Multiplicity of infection was 50 (**C**, **D**, **E**, **F**, **G**, **I**) or 100 (**A**, **B**, **H**). Data in (**A**, **D**, **G**, **I**) are combined from n=3 experiments. Data in (**B, E**, **F**, **H**) are representative from n=2-3 experiments. Data in (**C**) are combined from n=4 experiments. Significance was determined by one-way ANOVA followed by Tukey's post hoc test. Data are expressed as means ± SD. *P< 0.05, **P< 0.01, ***P< 0.001, ****P<0.0001

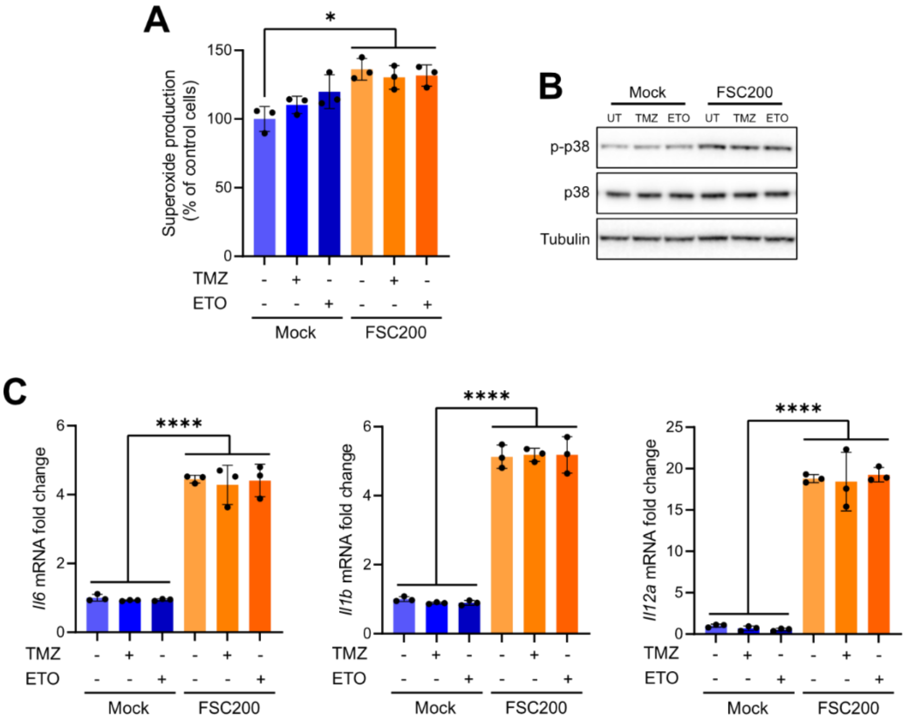

Fig. S6. Fatty acid β-oxidation does not fuel mtROS-dependent activation of *Francisella*-infected DCs *in vitro*. (**A**) Superoxide production, (**B**) p38 activation, and (**C**) *Il6*, *Il1b*, and *Il12a* expression in BMDCs pretreated (or not) with 10 µM trimetazidine (TMZ) or 5 µM etomoxir (ETO) for 1h and then infected for 1h (**A**, **B**) or 6h (**C**) with FSC200. *Actb* was used as a housekeeping gene in (**C**). Signal response in (**A**) is normalized to uninfected and untreated cells (=100%). Multiplicity of infection was 50 (**C**) or 100 (**A**, **B**). Data in (**A**, **B**) are representative from n=2 experiments. Data in (**C**) are combined from n=3 experiments. Significance was determined by one-way ANOVA followed by Tukey's post hoc test. Data in graphs are expressed as means ± SD. *P< 0.05, ****P<0.0001.

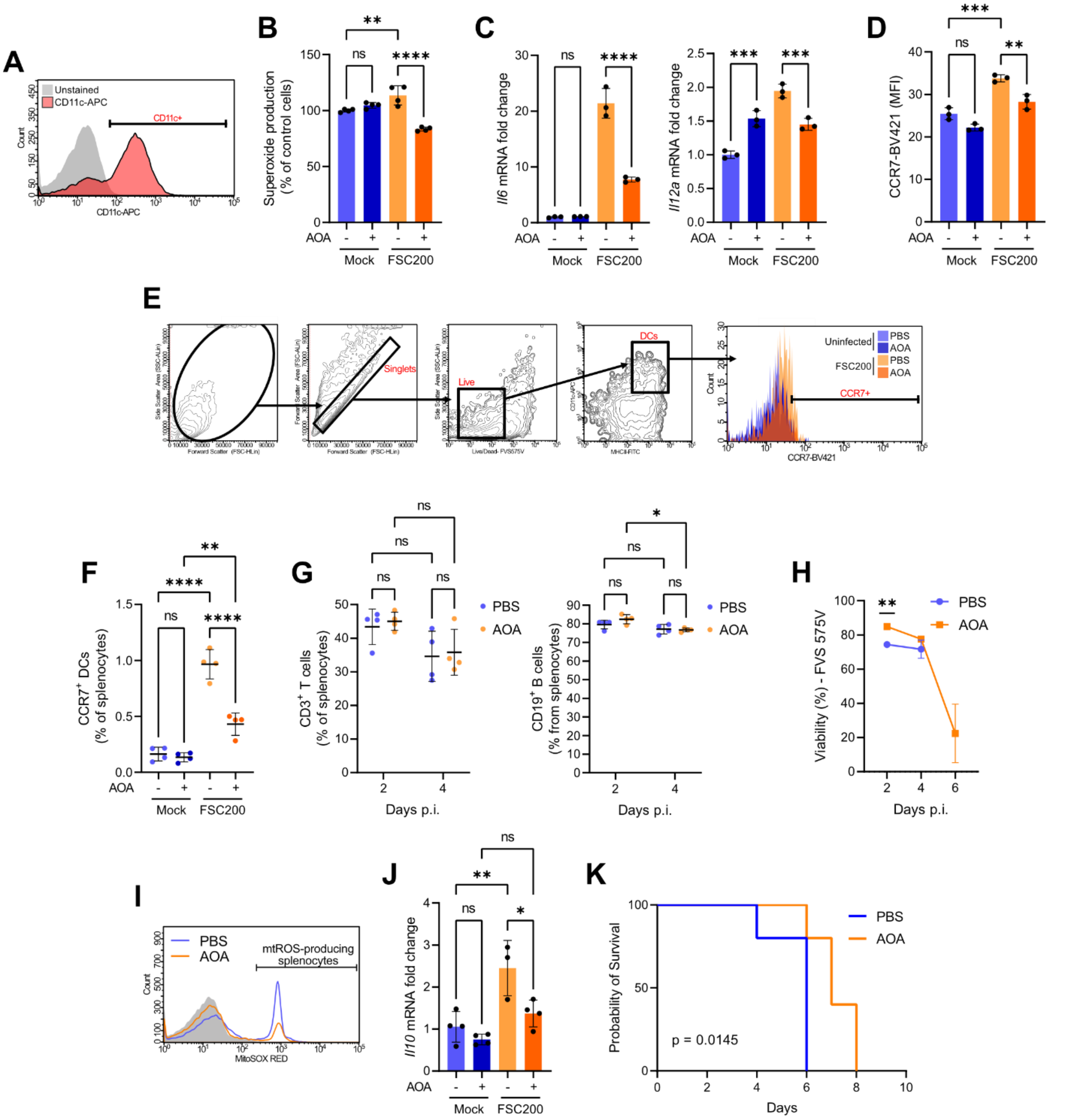

Fig. S7. Inhibition of glutamate metabolism *in vivo* attenuates the development of severe tularemia. (**A**) CD11c expression in Flt3L-derived BMDCs generated *in vitro*. Routinely, 75-85% cells were CD11c$^+$ positive. (**B**) Superoxide production, (**C**) *Il6* and *Il12a* expression, and **(D)** CCR7 cell surface expression in Flt3L-dependent BMDCs pretreated (or not) with 10 mM AOA for 1h and then infected for 1h (**B**), 6h (**C**), or 24h (**D**) with FSC200. *Actb* was used as a housekeeping gene in (**C**). (**E**) Gating strategy for flow cytometric analysis of splenic CCR7$^+$ DCs with representative histograms. (**F**) Frequency of migratory DCs (defined as CD11c$^+$ MHCII$^+$ CCR7$^+$) and (**G**) T cells (defined as CD3$^+$ CD19$^-$) and B cells (defined as CD3$^-$ CD19$^+$) within the

population of alive splenocytes isolated from spleens of FSC200-infected mice treated with PBS (mock) or AOA on day 2 and day 4 p.i. (n=4 mice per group). (**H**) splenocyte viability in FSC200-infected mice treated with PBS (mock) or AOA (n=4 mice per group except for day 6 p.i. (AOA) where n=2). All PBS-treated mice succumbed to infection before day 6 p.i. (**I**) Representative histograms from analysis of mtROS production in splenocytes on day 2 p.i. Related to **Fig. 6E**. (**J**) Expression of *Il10* in splenocytes isolated from uninfected or FSC200-infected mice treated with PBS (mock) or AOA on day 4 p.i. *Tbp* was used as a housekeeping gene (n=3-4 mice per group). (**K**) Survival of FSC200-infected BALB/c mice treated with PBS or AOA (n=5 mice per group). Mice were treated identically to C57BL/6 mice in **Fig. 6H**, based on scheme in **Fig. 6A**. Signal response in (**B**) is normalized to uninfected and untreated cells (=100%). For **B**-**D**, multiplicity of infection was 50 (**C**, **D**) or 100 (**B**) and the results are combined from n=3 (**C**) or are representative from n=2 (**B**, **D**) experiments. For *in vivo*, data in (**E**, **F**, **J**) are from one experiment and data in (**G**, **H**, **I**) are from another experiment of similar design (**Fig. 6A**). Data in (**K**) are from one experiment. Significance was determined by one-way ANOVA followed by Tukey's post hoc test (**B**, **C**, **D**, **F**, **J**), two-way ANOVA followed by Sidak's post hoc test (**G**, **H**), or by log-rank test (**K**). Data in graphs are expressed as means ± SD. $^*P < 0.05$, $^{**}P < 0.01$, $^{****}P < 0.0001$.

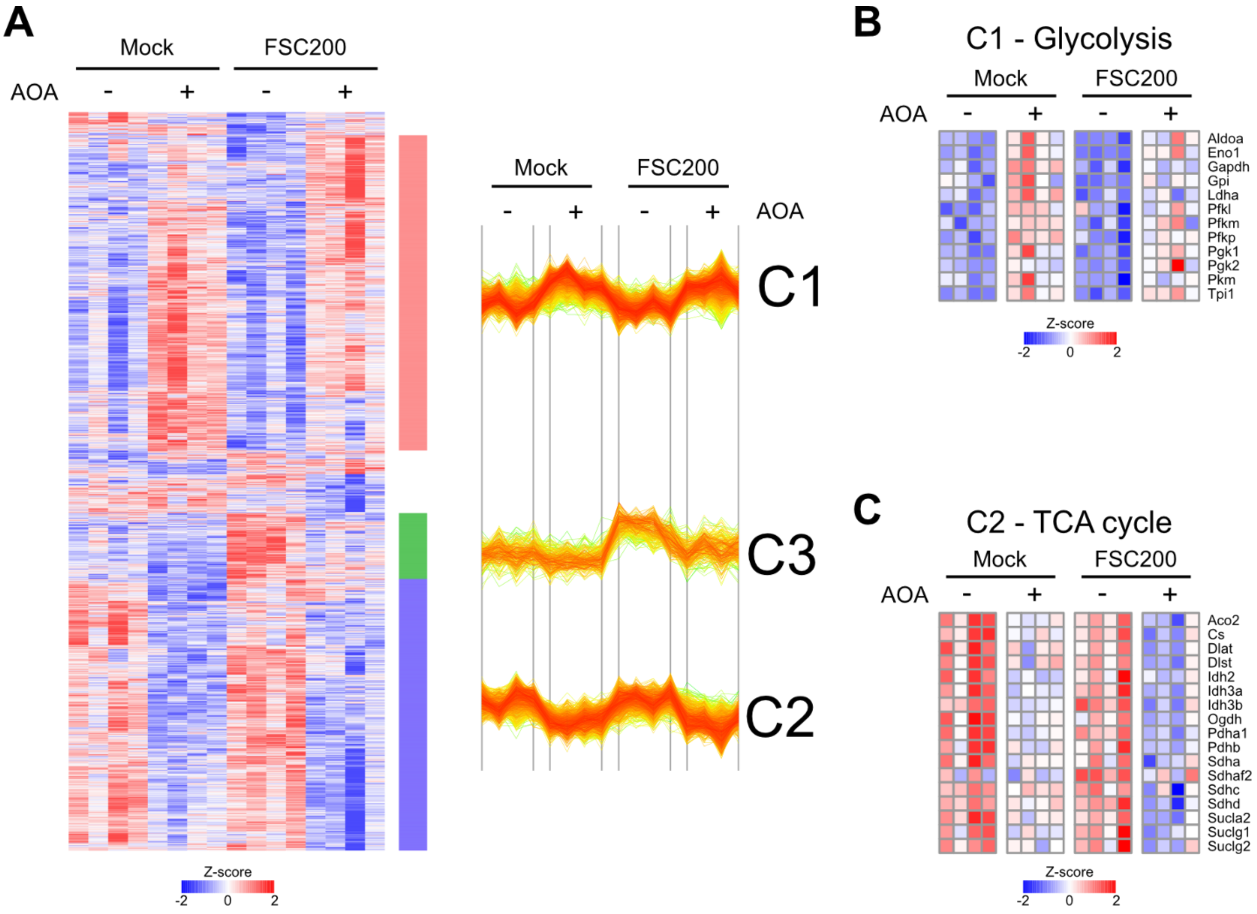

Fig. S8. Proteomic changes in splenocytes isolated from AOA-treated mice infected by *Francisella*. (**A**) Heatmap showing significantly regulated proteins (ANOVA, FDR<0.05) in splenocytes isolated from mice treated (or not) with AOA and infected (or not) with FSC200 on day 4 p.i. (n=4 mice per group). TMTpro reporter $\log_2$-intensities were transformed by Z-score and three clusters (C1, C2, C3) containing proteins with similar expression profiles were found by hierarchical clustering. (**B**) Expression of proteins annotated by GO term "Glycolysis" from cluster C1. (**C**) Expression of proteins annotated by GO term "Tricarboxylic acid cycle" from cluster C2. The experiment was done once.

Data S1. (separate file)
List of DC proteins co-immunoprecipitated with *Francisella* LPS

Data S2. (separate file)
List of proteins regulated by MitoTEMPO in *Francisella*-infected DCs

Data S3. (separate file)
List of protein acetylation (K) sites identified in *Francisella*-infected DCs

Data S4. (separate file)
List of proteins regulated by AOA in splenocytes isolated from *Francisella*-infected mice (day 4)

Data S5. (separate file)

Source Data
